# Improving hospital care for people who use drugs: deliberative process development of a clinical guideline for opioid withdrawal management

**DOI:** 10.1186/s12954-024-01127-2

**Published:** 2024-11-18

**Authors:** Marisha Wickremsinhe, Adam Holland, Jenny Scott, Rosalind Gittins, Michael Brown, Adrian ‘Bean’ Noctor, Dan Lewer, Vivian Hope, Niamh Eastwood, Magdalena Harris

**Affiliations:** 1 Department of Public Health, Environments & Society, London School of Hygiene & Tropical Medicine, 15-17 Tavistock Place, London, WC1H 9SH UK; 2 University of Bristol, Oakfield Grove, Clifton, Bristol, BS8 2BN UK; 3 Via, 18 Dartmouth St, London, SW1H 9BL UK; 4 University College London Hospitals NHS Trust, 235 Euston Road, London, NW1 2BU UK; 5 Department of Clinical Research, London School of Hygiene and Tropical Medicine, Keppel Street, London, WC1E 7HT UK; 6 Bradford Institute for Health Research, Bradford Royal Infirmary, Duckworth Lane, Bradford, BD9 6RJ UK; 7 Public Health Institute, Liverpool John Moores University, 3rd Floor Exchange Station, Tithebarn Street, Liverpool, L2 2QP UK; 8 Release, 61 Mansell Street, London, E1 8AN UK

**Keywords:** Opioid substitution treatment, People who use drugs, Methadone, Buprenorphine, Clinical governance, Policy, Patient-centred care, Hospitals

## Abstract

**Background:**

Management of opioid withdrawal in hospital settings is crucial to improve treatment completion and health outcomes among patients who use opioids, such as heroin. Evidence-based clinical guidelines can support responsive provision of opioid substitution therapy (OST). In England there is no standardised application of guidance for substance dependence management across National Health Service (NHS) Hospitals. A recent review of NHS hospital policies identified varying approaches to managing opioid withdrawal and procedural barriers to timely medication.

**Objective:**

To develop a clinical guideline for opioid withdrawal management in acute NHS hospital trusts to be tested and evaluated as part of the iHOST (Improving Hospital Opioid Substitution Therapy) research intervention.

**Methods:**

We undertook a deliberative guideline development process. The University London College Hospital (UCLH) substance dependence guideline was used as a template, with key points of revision informed by evidence review, consultations with hospital staff and people with opioid dependence. A multidisciplinary working group deliberated evidence statements to develop recommendations. These were reviewed by an oversight committee comprising representatives from key stakeholder organisations. The team authored the guideline with iterative review by the oversight committee, key stakeholders and UCLH clinical governance committees.

**Results:**

Deliberation focused on three key domains: (1) identifying opioid dependence and promptly continuing existing OST prescriptions; (2) initiating or re-titrating OST; (3) ensuring safety and continuity of care at discharge. Changes to the UCLH guideline included removal of mandatory urine drug testing prior to OST; increasing initial methadone titration dose; and provision for a higher day-one titration dose when specific safety criteria are met. A new titration schedule for sublingual buprenorphine was incorporated. Discharge planning to ensure continuity of community care and reduce risk of opioid overdose was emphasised, with allowance for bridging prescriptions of OST and naloxone provision on hospital discharge.

**Conclusion:**

The iHOST clinical guideline aims to remove procedural barriers to opioid withdrawal management for hospital inpatients. It is intended to be implemented by other NHS hospitals, which could improve access to OST and reduce discrepancies in treatment access and completion.

**Study registration:**

ISRCTN47320412 10.1186/ISRCTN47320412.

**Supplementary Information:**

The online version contains supplementary material available at 10.1186/s12954-024-01127-2.

## Background

People who use opioids such as heroin face high risks of morbidity and mortality. Drug-related deaths are at record levels across the UK [1–3], and people who use illicit opioids are more likely to die from all causes of death when compared with people of the same age in the general population [4]. This group experience higher rates of emergency hospital admission [5], often to address chronic health issues, injuries, and infections [5–9]. They may delay seeking treatment [10, 11] and leave hospital before treatment completion [12], resulting in emergency readmission, more expensive and complicated medical care, and a greater risk of all-cause mortality [13–16].

Qualitative research demonstrates that fear of opioid withdrawal in hospital is a key barrier to seeking and completing treatment [11]. Experiences of opioid withdrawal as an inpatient can cause physical and psychological distress leading to self-discharge, ward absences, and disrupted treatment as patients leave hospital to source opioids [12, 16–21]. Hospital discharge is a key risk period for fatal opioid overdose, attributable to reduced opioid tolerance following insufficient opioid substitution therapy (OST) provision during admission and the use of additional sedatives to manage symptoms of an acute illness [22, 23]. Other issues which impact patients’ willingness to engage with hospital care include poor pain management, restrictions on behaviour during admission, and experiences of stigma [11, 24, 25]. Both Dame Carol Black’s Independent Review of Drugs [26] and the 2021 UK Drugs Strategy [27] highlight the need to improve physical healthcare for people who use drugs.

In the UK, OST—usually methadone or buprenorphine—is recommended for people experiencing opioid dependence [28]. Timely and effective provision of OST in hospital is critical to adequately manage opioid withdrawal symptoms, which can, in turn, ensure treatment is completed for presenting medical conditions. OST should be prescribed in hospital either to continue care received in the community, if the patient is already on an OST prescription, or to prevent opioid withdrawal if the patient uses illicit opioids such as heroin and is not on an OST prescription.

Given the importance of OST provision in hospital, national guidelines for the clinical management of drug dependence, authored by the UK Department of Health, recommend that hospitals implement local protocols to facilitate prompt OST provision on admission and to enable continuity of care on discharge [29]. Our published document analysis of policies across 86 acute NHS hospital trusts in England demonstrates procedural barriers to prompt OST provision and significant variability in recommended approaches [30]. These policies often differed from national guidance, which itself draws on a limited evidence base [29]. Obstacles to OST provision in hospital include: (i) limited guidance on how to confirm routine OST prescriptions when community services are closed; (ii) restrictions on OST dosing during initiation; (iii) insufficient guidance for OST continuity on discharge; (iv) procedural barriers, particularly requirements for positive drug tests prior to OST prescription; and (v) negative characterizations of people who use opioids [30]. Policies typically emphasized the risk of opioid toxicity from OST administration with little acknowledgment of risks associated with insufficient OST provision, including intolerable withdrawal symptoms, self-discharge, and incomplete treatment [30]. The reason for this level of variability in hospital policies is not clear. Hospitals are responsible for developing their own policies (while not being required to follow national guidance). Differences may reflect relative levels of awareness and expertise in the management of opioid dependence, without a definitive evidence base to inform policy development, as well as the recency and thoroughness of policy revision.

Here, we detail the process and outcomes of revising the opioid withdrawal management guideline at University College London Hospital (UCLH) to address procedural barriers identified through our document analysis [30]. UCLH is a major teaching hospital in central London. Clinicians across medical and surgical specialties manage opioid dependence and withdrawal with support from drug and alcohol nurse specialists. When required, additional support may also be provided by colleagues from liaison psychiatry and community drug treatment services. The guideline revision was conducted as part of a broader project—the National Institute for Health and Care Research (NIHR) funded Improving Hospital Opioid Substitution Therapy (iHOST) study [31]—which aims to inform improvements to OST provision in acute NHS hospitals nationally.

## Methods

The iHOST study seeks to develop and evaluate a multi-component intervention consisting of: (1) a patient-centred ‘best practice’ clinical guideline for opioid withdrawal management in hospital to enable timely and effective OST provision; (2) a patient advocacy card (‘MyMeds’) to empower people on a community OST prescription to access OST in hospital and facilitate faster confirmation of dosage; (3) an OST helpline run by the drug policy charity, Release, to support admitted patients in advocating for access to OST; (4) a hospital champion role to support implementation of the iHOST intervention; and (5) an e-learning module for hospital staff to reduce stigma and improve knowledge of opioid dependence and OST. In this paper, we report on the development process for this first component of the intervention, the iHOST guideline. The guideline development process comprised the following steps:

### Consultation with UCLH staff and NHS patients

We consulted UCLH staff and people who use opioids to identify how hospital guidelines could support prompt and effective management of opioid withdrawal in hospital. Between October 2021 and April 2022, MH conducted four focus groups with people with lived and living experience of opioid dependence and hospital admission (total *n* = 16 across the four groups). Two of these focus groups were conducted with people who use opioids recruited by MH through personal and professional networks, two were conducted in collaboration with local specialist drug services. MH used a topic guide aimed at understanding participants experiences of hospital admission, OST access, opioid withdrawal and hospital discharge. The groups were audio recorded with written participant consent, transcribed verbatim and thematically analysed using an inductive approach.

In March 2022, MH and JS conducted a focus group with 14 UCLH staff members, including nurses, junior doctors, and consultants. Staff were invited to participate by MB, a Consultant Physician at UCLH. MB audited OST prescription practices across the hospital and involved staff who worked on wards where OST is commonly prescribed. The group was informed by a topic guide aimed at understanding current OST prescribing practices for inpatients, staff experiences of managing opioid withdrawal, and barriers and facilitators to effective withdrawal management. The group was not audio-recorded, given some staff movement in and out of the session. MH and JS took detailed anonymised notes of staff responses with participant consent. These were reviewed and thematically categorised.

In April 2022, JS conducted individual consultations with two senior UCLH pharmacists. During these consultations a diagram was created of the process of OST prescription and administration. Then a root cause analysis approach was taken [32], including data from the focus groups and pharmacist consultations, to identify points at which delays or barriers to OST could be created and why these occurred. These were compared with findings from our previously published review of existing NHS hospital opioid withdrawal policies [30] to highlight where guidance posed the most significant barriers to prompt and effective withdrawal management.

### Deliberative guideline development process

To develop the guideline recommendations, we undertook a deliberative process approach, which “provides guidance informed by relevant scientific evidence, interpreted in a relevant context wherever possible with context-sensitive scientific evidence and, where not, by the best available colloquial evidence” [33]. ‘Colloquial evidence’ refers to any information other than findings from scientific research studies, including information about resources, context, values, and particularly the opinions and practical experience of clinical and patient experts [34, 35]. In practice, a deliberative process requires that an inter-disciplinary group of experts iteratively appraise the appropriateness of different courses of action, considering the advantages and disadvantages of their likely intended and unintended consequences, reflecting on available evidence and others’ views [33].

Deliberative processes are widely used in health policy decision-making, including the Health Technology Assessments undertaken by the UK National Institute for Health and Care Excellence (NICE) [34]. This approach is considered suitable where (a) multiple types of evidence are relevant (such as clinical guidelines for related conditions, clinical expertise, and patient views) and (b) where guidance must be sensitive to matters of equity [33]—both relevant to the context of the iHOST guideline development process.

Given the relative lack of empirical evidence informing appropriate OST regimens in hospital settings, a deliberative process was necessary to interpret and contextualize evidence developed in other contexts, which has unclear external validity relative to the hospital setting; and to draw heavily on existing guidance. The deliberative process enabled us to consider not only the risks of opioid withdrawal and toxicity in hospital, but also the health and social risks associated with self-discharge due to untreated opioid withdrawal. With this approach, we centred equity considerations, given the discrimination faced by people who use drugs, and the lack of parity between guidelines informing medical care for this group as compared to other patient populations.

The deliberative process included an evidence review, workshops and consultations with experts. The process was led by the iHOST team in collaboration with (i) the iHOST Policy Template Working Group; (ii) the iHOST Policy Template Oversight Group; and (iii) additional experts who were individually consulted. Each group was engaged concurrently to enable reflection and feedback on differing perspectives between groups, as communicated by the iHOST team. Key evidence-gathering activities, membership of groups for consultations, and specific engagement processes are detailed below.

### National guidance and other evidence summaries

We reviewed *Drug misuse and dependence - UK guidelines on clinical management* (often called ‘The Orange Book’), authored by the UK Department of Health [29]; the NICE technology appraisal, ‘Methadone and buprenorphine for the management of opioid dependence’ [28]; and British National Formulary (BNF) prescribing guidelines for methadone [36] and buprenorphine [37]. MW, AH and MH extracted and compared guideline data regarding dose titration and OST initiation protocols, opioid dependence assessment, and hospital-specific guidance, where available. Relevant text extracts from these guidelines, qualitative data analyses from staff and patient/client focus groups, findings from our review of NHS substance dependence policies [30], and other appropriate evidence, were summarized and provided to the Working and Oversight Groups prior to workshops to support discussion. For example, inconsistencies between guideline sources (e.g., different recommendations for maximum doses of methadone on days one and two of titration) were highlighted for discussion with stakeholders, alongside excerpts from each guideline on titration dosing schedules, data from our review of NHS hospital policies, and qualitative data from people who use drugs and hospital staff regarding their experience of titration practice.

### iHOST policy template working group

The Working Group was tasked with considering the benefits and risks of potential recommendations for the iHOST guideline, focusing on specific points of contention identified in our reviews of national guidance, local NHS policies, and consultations with staff and patients. This group comprised 12 members, including people with lived experience of opioid dependence as well as doctors, nurses, pharmacists, and drug law specialists. Members were invited to participate based on their expertise and prior work in addiction medicine and related fields. They were recruited through existing networks of the iHOST study team and project partners.

Five one-hour videoconference workshops were held between June and August 2022, facilitated by MW. The first four workshops focused on: (i) drug testing prior to prescription; (ii) provision of takeaway OST medications on discharge; (iii) medicines reconciliation on admission for patients prescribed OST in the community; and (iv) discharge planning protocols. Following agreement on key parameters, the final workshop focused on review and finalization of the new guideline’s flow diagram.

### iHOST policy oversight group

The Oversight Group was tasked with reviewing and providing feedback on draft recommendations developed by the iHOST team in collaboration with the Working Group. To identify key members to include in the group, AH conducted a stakeholder analysis with input from the wider iHOST team, characterizing the likely interest and influence of candidate organizations from which to recruit representatives. Organizations were selected based on these factors as well as their expertise. Six organizations agreed to join the group: Addiction Professionals, the British Pharmacological Society, the College of Mental Health Pharmacy, the Office for Health Improvement & Disparities (OHID), the Royal College of Psychiatrists and the Royal Pharmaceutical Society. The individuals who represented the organizations either self-appointed following initial contact or were nominated through internal organizational procedures.

Two two-hour videoconference workshops were held with the Oversight Group in July and August 2022, facilitated by MW, AH, and MH. Prior to each workshop, Oversight Group members also offered written feedback on iterative drafts of the guideline authored by the iHOST team. MW synthesized this feedback to organize workshop discussions. In both sessions, discussions focused on key areas of contention, supported by ‘rationale statements’ summarizing potential risks and benefits of proposed recommendations, alongside relevant consultation findings, national and local guidance and available evidence. Following each workshop, the iHOST team provided detailed responses to each Oversight Group member’s written comments. A summary of discussions was presented to the Group to ensure that all revisions to the draft guideline were captured systematically. All group members were provided with a finalized draft of the guideline, as were additional expert stakeholders affiliated with the Addiction and Inclusion Directorate of OHID, and clinicians and pharmacists employed by UCLH.

### Clinical governance review at UCLH

The guideline was submitted for approval through standard guideline ratification processes in UCLH. It was formally reviewed by three of the Trust’s established clinical governance bodies: the Opioid Stewardship Committee, the Use of Medicines Committee, and the Clinical Guidelines Committee. At each stage, the guideline was presented in committee, discussed, and amended in line with feedback to ensure that the recommendations were appropriately contextualized to local needs, including workloads of relevant staff. The final version of the guideline was signed off by the Controlled Drugs Accountable Officer and the Medical Director and was implemented as the UCLH Trust Wide Guideline in November 2022.

### Ethics approval

The iHOST study received ethical approval from NHS Ethics (IRAS 310856) and the London School of Hygiene & Tropical Medicine (Ref 27895). Participants who use opioids were reimbursed in cash payments for their time and expertise. Hospital staff were not reimbursed as they were consulted during normal working hours.

## Results

We report decision-making on guideline parameters across three key domains: (i) identifying opioid dependence and continuing OST prescriptions; (ii) initiating or re-titrating OST in hospital; and (iii) ensuring safety and continuity of care on discharge. While the scope of the guideline is broader, covering issues such as contraindications to OST provision and pain management, these three domains were the most contentious and thus the prime focus of the deliberative process.

### Identifying opioid dependence and continuing OST prescriptions

The guideline sought to facilitate timely and effective OST provision in hospital, by ensuring that patients with opioid dependence are promptly and correctly identified through medical assessment. This included review of medicines reconciliation protocols for patients already in receipt of community OST. Prior UCLH guidance required a positive urine drug screen (UDS) prior to OST prescription, regardless of whether the patient was on a confirmed community prescription or was clearly showing signs of opioid withdrawal. Staff consultations highlighted practical challenges imposed by this requirement, especially given the long delays caused by laboratory testing. However, they also expressed reservations about using point-of-care (POC) testing, given prior experiences of a POC pilot scheme which highlighted constraints related to staff training and capacity. From the perspective of people who use opioids, our consultations found that UDS can exacerbate stigma and foster distrust, undermining therapeutic relationships.

Requirements for UDS in hospital guidelines are variable across England. Of the trust policies reviewed, around half did not require or recommend UDS prior to OST prescription, whereas 16% required a positive result prior to any OST provision, even if the patient was on a confirmed community prescription [30]. National guidelines include UDS as one means of verifying recent drug use while also highlighting the risk of false negatives and that the tests cannot confirm dependence or tolerance [29].

During the deliberative group process a range of opinions were expressed regarding use of mandatory UDS prior to OST prescription. Arguments in favour of requiring a positive UDS included their perceived objectivity to corroborate patients’ self-reported opioid use, given the risk of toxicity if the patient incorrectly reports use and has lower than expected tolerance. Some suggested that UDS could be a useful tool for clinicians who are less experienced in assessing opioid dependence and withdrawal and mitigate medicolegal risks of opioid prescribing. Arguments against a UDS requirement included the clinical duty to promptly treat observable opioid withdrawal symptoms without waiting for screening results. Further, some argued that reliance on UDS could lead to false assurance among clinicians, as a positive test provides evidence only of recent opioid use, not of dependence or tolerance. The risk of patients leaving hospital due to withdrawal while waiting for test results was noted as a concern. This risk needed to be weighed against perceived medicolegal risk – which some argued could be mitigated by obtaining patient consent prior to OST prescription. After this deliberative process, consensus was broadly reached that, while UDS is of clinical use in specific circumstances, a mandatory UDS requirement prior to OST prescription can increase risk of patient self-discharge and be unduly onerous for staff, particularly when POC testing is not available.

The iHOST guideline therefore acknowledges that UDS may be clinically useful (e.g., to support further discussion of a patient’s drug use) but clarifies that evidence of opioid dependence gained through a patient history, medical records, and structured clinical observation is sufficient to prescribe OST with patient consent. To identify patients with opioid dependence, the guideline does not require a positive UDS prior to OST prescription but instead emphasizes the importance of taking a thorough history and withdrawal observation. Furthermore, the guideline clarifies that a patient’s refusal to provide a urine sample should not delay OST provision. Where a UDS is pursued, the guideline highlights the need to explain the purpose of the test and obtain patient consent.

To support clinicians in taking a comprehensive drug history and assessing opioid withdrawal, the guideline includes an appendix highlighting key questions, with suggested language to support clinicians to communicate clearly and respectfully. The appendix also includes a copy of the Clinical Opioid Withdrawal Scale (COWS) [38] to support staff in assessing withdrawal symptoms.

To continue community OST prescriptions in hospital, prior UCLH guidance required clinicians conduct a UDS and to contact both the community prescriber and pharmacist to verify the dose and date of last consumption or pick-up. UCLH staff reported that this dual-verification overly burdened staff time, both in the hospital and community. Discussions within the Working Group highlighted that this requirement is inconsistent with protocols for other medicines, where the patient report can be confirmed by one additional source, usually a healthcare professional. Through the deliberative process with both groups, a decision was made that only one other source should be required to confirm a patient’s routine OST dose (except where the patient lacks capacity, in which case two independent sources would be required as is standard practice in medicines reconciliation when patient capacity is in question). The iHOST guideline therefore states that confirmation of the dose and date of last consumption or pick-up is required from only one further source, in addition to the patient, for anyone admitted on a community OST prescription. While both the community prescriber and the community pharmacist should be informed of the patient’s admission, hospital staff can prescribe a patient’s routine OST following dose confirmation. The Working Group and Oversight Group also discussed other methods of confirming the patient’s routine dose where contact with the community pharmacist or prescriber is not possible (e.g., out-of-hours). The guideline includes guidance on these alternative valid sources, including a labelled and recently issued OST bottle or prescription.

For patients on a community prescription for methadone, the iHOST guideline notes that, even where the community dose has been confirmed and prescribed as usual, patients should be routinely monitored for withdrawal symptoms. Where symptoms persist, a ‘when required’ or *pro re nata* (PRN) dose of 5 mg to 10 mg of methadone can be prescribed to account for withdrawal attributable to ‘on-top’ use of other opioids in the community. Prescription of other medications for opioid dependence is uncommon in the UK, but can include injectable medications, and depot buprenorphine. We indicate in the guideline that if individuals who are taking these medications present to hospital, their management should be discussed with the local drug service provider.

### Initiating or re-titrating OST in hospital

The guideline sought to enable prompt and effective OST initiation in patients who are not on a community prescription, and to provide clear guidance on re-titrating OST in patients on a community prescription that cannot be confirmed (e.g., when admitted out-of-hours). For these patients, the prior UCLH guidance we were amending only outlined a titration schedule for methadone, explicitly advising that buprenorphine should not be initiated. Methadone dosing was indexed to COWS score (between 5 mg and 30 mg according to symptom severity), with additional methadone prescribed and administered four-hourly, up to a maximum dose of 40 mg of methadone on day one.

UCLH staff highlighted challenges with this protocol, especially where the patient’s withdrawal symptoms were classified as too mild to warrant a methadone dose that patients deemed adequate. Staff felt that low initial doses were more likely to lead patients to leave hospital to self-treat withdrawal. Likewise, people who use opioids suggested that lower initial doses of methadone were often inadequate to alleviate withdrawal symptoms. Both UCLH staff and people who use opioids highlighted that doses of methadone above 40 mg may be required in some cases to prevent withdrawal.

Our document analysis of NHS hospital policies demonstrated significant variability in methadone dosing [30]. Recommended starting doses ranged from 5 mg to 40 mg, and total day-one maximum doses from 20 mg to 70 mg. This ambiguity is compounded by the differing recommendations in national guidance. For methadone administered in hospital, Department of Health guidelines recommend a starting dose of 10 mg, to be increased by 10 mg four-hourly up to a maximum total of 40 mg on day one [29]. However, for methadone prescribed in community settings, the national guidelines recommend a starting dose of 30 mg, with allowance for up to 40 mg under specific circumstances [29]. The latter is consistent with dosing recommendations of the BNF [36].

Given discrepancies between existing local and national guidelines, we discussed initial dosing with the Working Group and Oversight Group. To scaffold discussion, the iHOST team asked both groups to contrast risk/benefit analyses of a 10 mg, 20 mg, or 30 mg initial dose of methadone in hospital. A key argument against higher initial doses was the risk of toxicity. Arguments in favour of higher initial doses were reducing the risk of self-discharge, preventing patient self-medication to treat withdrawal symptoms, and enabling patients to stay in hospital. Deliberations across both groups concluded that 10 mg of methadone is unlikely to be of sufficient benefit to patients experiencing opioid withdrawal, which could increase the likelihood of self-discharge. It was felt that some patients may require more than 40 mg to prevent withdrawal, however that prescriptions totalling more than 40mgs on day one should only be permissible in specific cases under expert supervision due to toxicity risk.

The iHOST guideline acknowledges that the hospital setting can be less risky for initiating or re-titrating OST as compared with the community setting, assuming appropriate staffing levels and monitoring capacity to allow routine observation. Therefore, it recommends a starting dose of up to 20 mg of methadone for patients experiencing withdrawal, with consideration of 30 mg in the case of severe symptoms. Thereafter, the guideline recommends prescribing further doses titrated against withdrawal symptoms up to 40 mg in total on day one.

In very specific circumstances, the guideline allows for an increase in the total day-one dose of methadone from 40 mg to 60 mg. This dose increase should be considered only where the patient is still experiencing severe withdrawal symptoms after 40 mg of methadone. The guideline highlights this decision must be taken by a senior decision-maker, with specified examples: a drug and alcohol clinical nurse specialist, specialist pharmacist, senior medical clinician or anaesthetist. Furthermore, the guideline highlights that a day-one dose higher than 60 mg should only be considered when close nursing supervision is available. To mitigate risks of methadone toxicity, the guideline mandates routine monitoring of respiratory rate, oxygen saturations, and consciousness level; the availability of naloxone in ward stocks for emergency management; and that naloxone be prescribed PRN. Guidance is provided on management of overdose and thresholds for clinical escalation.

In the UK context, methadone is more commonly prescribed to manage opioid dependence than buprenorphine, but prescription of the latter is becoming more common [39]. Our review of NHS policies found that buprenorphine was not referenced at all in 22% (19/86) of guidelines reviewed, and only 27% of trusts (23/86) included a buprenorphine initiation schedule [30]. The iHOST guideline offers advice on choosing between methadone and buprenorphine (with reference to relevant clinical considerations, patient preference, medicine availability, and pain management needs). A dosing regimen for buprenorphine in line with national clinical guidance is recommended, which should only be commenced when withdrawal symptoms are present to avoid precipitated withdrawal [29].

### Ensuring safety and continuity of care on discharge

The iHOST guideline sought to improve continuity of care and ensure safety for all patients receiving OST discharged from hospital. Prior UCLH guidance offered limited advice on continuing OST on discharge. The guideline also prohibited the provision of takeaway OST doses and made no provisions for takeaway naloxone.

UCLH staff suggested that the lack of support around discharge planning could lead to patients being admitted for longer than medically necessary, exacerbating bed pressures. These extended stays were most likely when continuation of OST could not be arranged with community services, e.g., if the patient was deemed medically fit for discharge over the weekend. Conversely, in other cases, people with opioid dependence may be discharged without continuity of care, placing them at risk of self-medicating withdrawal symptoms in unsafe settings. Consultations with people who use opioids confirmed these concerns. A number reported experiences of being discharged without access to OST, in some cases leading them to use heroin while at greater risk of overdose due to reduced tolerance. Accordingly, we considered the merits of providing OST doses on discharge from hospital if community services were not able to ensure continuity of care. Our review of NHS policies found that 66% (57/86) of trusts permitted takeaway OST in some circumstances [30]. National guidelines also permit a small number of takeaway OST doses (usually one to three days) on discharge to facilitate continuity of care for patients who were admitted on a community OST prescription [29].

Both the Working Group and the Oversight Group emphasized the importance of early discharge planning to mitigate the risk of OST interruption. In terms of takeaway OST doses, opinions varied. Arguments in favour of takeaway OST doses included the need to mitigate the elevated risk of overdose following hospital discharge, potentially attributable to illicit opioid use in the context of OST interruption [22]. Arguments against providing takeaway OST included concerns about diversion (where the patient shares OST medications with other people). Some suggested that instead of supplying takeaway OST, a priority appointment should always be arranged with community services for the day of discharge. However, implementing this recommendation would necessitate limiting hospital discharge to times when community drug treatment services are open (usually Monday to Friday during working hours), which is infeasible given resource constraints. The Oversight Group therefore suggested that, at least in some cases, takeaway OST may be appropriate to enable continuity of care, specifically for patients admitted on a community prescription and where the provision of takeaway doses has been agreed with the community prescriber prior to planned discharge. There was unanimous agreement across the Working Group and Oversight Group that all patients prescribed OST in hospital should be provided naloxone on discharge.

The iHOST guideline, therefore, stresses the importance of early discharge planning to ensure that the community drug treatment service is aware of the patient’s admission and kept informed of any changes made to patient OST prescriptions in hospital. The guideline permits takeaway OST doses for one to three days to ensure continuity of care for people discharged out-of-hours who are already on a community OST prescription if agreed with the community provider. Additionally, it recommends the provision of takeaway naloxone to all patients prescribed OST in hospital alongside education on how it should be used in the event of an overdose. Multiple training sessions on take home naloxone were delivered to staff to facilitate delivery and relevant resources including a training checklist are provided in the appendix of the guideline.

## Discussion

In this paper, we outlined the deliberative process used to develop the iHOST guideline. We incorporated the views of people who use opioids, hospital staff and clinical experts to ensure that recommendations were sensitive to relevant risks, including self-discharge, post-discharge overdose, and inpatient opioid toxicity. The primary objective of the guideline is to ensure timely management of opioid withdrawal symptoms, thereby supporting inpatients to remain in hospital and complete treatment. As the guideline is eventually intended for use across NHS hospitals, without additional resources and where specialist clinicians may not be available, the recommendations focus on care that can be provided by non-specialist providers.

The guideline development process was informed by three guiding principles to improve care for inpatients who use opioids or receive OST. First, we sought to rebalance perceptions of risk, weighing not only the risk of OST toxicity, but also the risk of delaying or omitting OST, which can lead to intolerable withdrawal symptoms, self-discharge, and post-discharge overdose [11, 18, 21, 23]. Although the risks of not administering OST may be less immediately tangible to prescribing clinicians, these risks are of central importance to patients and their safety outside of the hospital. Therefore, the guideline emphasizes the importance of doses sufficient to relieve or prevent withdrawal symptoms, thereby enabling the patient to stay in hospital and complete treatment, while also reinforcing the need for careful monitoring and prompt treatment in the event of toxicity. The guideline additionally attends to post-discharge overdose risk by offering takeaway naloxone on discharge, and ensuring OST is provided while in hospital to prevent loss of tolerance.

Second, to achieve parity with guidance intended for other patient groups, we sought to engage people who use drugs in the development process. Patient involvement is widely understood to be a foundational component of guideline development [40]. However, no available NHS hospital opioid dependence policies included any reference to patient and public involvement [30]. We involved people who use drugs throughout the development process, including through membership of the Working Group.

Third, we sought to challenge stigmatising discourses and assumptions about people who use drugs, found in NHS substance dependence guidance [30], which can perpetuate and reinforce negative attitudes towards people who use drugs by healthcare workers [41]. We sought to promote patient-centred approaches that involve people who use drugs in decisions and information sharing related to their care. This contrasts with measures which implicitly or explicitly deny the veracity of patient testimonies, which treat people who use drugs as untrustworthy and without agency. One important aspect of reducing stigma towards people who use drugs is addressing how language is used [42]. We drew on guidelines developed by organizations representing people who use drugs [43] to inform language choices, with an emphasis on using neutral and person-first terminology. Implicit judgements regarding drug use, as present in terms such as ‘abuser’, ‘misuser’ or ‘addict’, for example, are problematic. Terms such as ‘substance use’ and ‘people who use heroin’ remove inferences of moral judgement and emphasise drug use as a practice (among many), rather than as an identity. These considerations informed our recommendations on appropriate and non-stigmatising approaches to take a drug history, included as a guideline appendix.

Most other research into improving hospital-based OST has been done in the United States. These interventions are primarily aimed at improving access to OST in the community, using hospital admission as an entry point to care, rather than preventing opioid withdrawal in hospital, which is the focus of iHOST. Some other relevant studies have evaluated models of addiction care for hospital inpatients. Several different models have been identified including addiction consult services, in-reach by community addiction teams, and staff training [44, 45]. These existing models informed development of the multicomponent iHOST intervention.

To our knowledge, there is limited evidence aside from patient case reports [46, 47] to guide specific details of OST management in hospital settings such as titration regimes. This may contribute to the disparities in hospital policies for opioid dependence management across the UK [30]. This is similarly the case for much guidance in the USA, as highlighted by a recent systematic review [48]. Since the iHOST guideline was developed, North American research has demonstrated that increasing the rate of methadone titration in hospital can be well tolerated by patients [49, 50]. However, it is important to note that these studies were conducted in the context of widespread adulteration of heroin supplies with fentanyl and other synthetic opioids. This is not currently the case in the UK, although it is becoming more of an issue, with an increasing number of deaths associated with potent nitazene opioids [51, 52]. In a context of increasing drug market toxicity, specialist drug services may need to innovate to retain clients and reduce their overdose risk. Any changes to community OST provision will be monitored and inform recommendations for commensurate change in iHOST hospital guidelines.

### Limitations

Given the lack of evidence to inform hospital specific recommendations, it was necessary to draw heavily on existing guidance and expert opinion, as has been the case for previous related guidance in the NHS [30] and internationally [48]. Accordingly, we adopted a deliberative process approach as described. It is important to note that there is limited evidence for deliberative process as a specific method to develop guidelines [34]. Success is likely to be influenced by the cadre of experts involved, and the specifics of the process ensuring that information is appropriately shared with and between stakeholders to allow informed decision making. The nature of the participants involved in the iHOST development process – including leading experts and clinicians in the UK – and meticulous efforts to ensure stakeholder views were appropriately shared and addressed were positive aspects of the deliberative process in this instance.

While a Delphi study or other formal consensus exercise would generally be preferable in the absence of context-specific evidence, this approach was not practicable given the time frame and scope of guidance under consideration. To account for the limited evidence on which we could draw to inform recommendations, and to monitor for any unintended consequences, a mixed methods evaluation of guideline implementation and concurrent safety monitoring is underway, which will subsequently be reported in the academic literature.

### Future work

While clinical guidelines play a critical role in improving OST provision in hospital [53, 54], guideline changes alone do not necessarily change practice or outcomes. For instance, a USA study found that revising an opioid withdrawal policy was associated with increased OST provision in hospital but no change in premature discharge [53] and a UK study highlighted inconsistencies in the implementation of a new opioid withdrawal guideline [54]. This demonstrates the need for complex interventions with multiple components, including wider measures to improve patient experiences, and staff training to ensure changes in practice.

Accordingly, the iHOST guideline is part of a wider multi-faceted intervention, with five components designed to address a range of factors critical to improving OST provision in hospital: (i) the guideline; (ii) staff training; (iii) a staff champion role; (iv) a patient helpline; and (v) an advocacy card (the My Meds card) for patients to carry highlighting the importance of receiving OST. While the guideline seeks to reduce procedural barriers to OST provision, the other components of the intervention seek to empower patients to advocate for access to OST (the My Meds card and the helpline) and to enhance staff knowledge and confidence in providing care for people who use drugs (the training and champion role). After iHOST implementation at UCLH (from November 2022) the intervention is being formally evaluated in two NHS teaching hospitals located in Leeds and Staffordshire. If found to be beneficial, the team will work towards informing national guidance to optimize and standardise care across acute NHS hospital trusts.

## Conclusion

The iHOST guideline for opioid withdrawal management, developed through a deliberative process in collaboration with expert stakeholders including people who use drugs, is now implemented as policy at UCLH. The guideline, and the wider iHOST study, aims to improve patient-centred care and support hospital provider confidence in effectively treating opioid withdrawal and providing continuity of care for people receiving community opioid substitution therapy. Along with the broader iHOST intervention the guideline provides a first step towards improving the confidence of people who use drugs in hospital care and ideally, in time, reducing their experiences and perceptions of prejudicial treatment. If appropriate for standardisation across NHS acute hospital trusts the guideline could help reduce discrepancies in treatment access and completion to improve health outcomes for a marginalised high-risk population.

## Electronic supplementary material

Below is the link to the electronic supplementary material.

Supplementary Material 1

## Data Availability

The iHOST/UCLH guideline is included as a supplementary information file. The team are monitoring any implementation of the iHOST guideline (full or partial). Please contact magdalena.harris@lshtm.ac.uk with any feedback, particularly regarding impact on practice.
