# Supplementary material for: Improving hospital care for people who use drugs: deliberative process development of a clinical guideline for opioid withdrawal management
Source: Harm Reduct J. 2024 Nov 18;21:201. doi: 10.1186/s12954-024-01127-2 (PMC11572091; doi:10.1186/s12954-024-01127-2)
Supplement: Supplementary file 1 — Supplementary Material 1 [file 12954_2024_1127_MOESM1_ESM.pdf]

uclh

Managing opioid withdrawal for inpatients with substance dependency

UCLH Guideline

Trust Wide

**Author(s)**	Mike Brown, Consultant Infectious Diseases<br>James Norman, Inclusion Health Consultant<br><br><u>Authors external to UCLH</u><br>Magdalena Harris, Associate Professor, London School of Hygiene and Tropical Medicine<br>Adam Holland, Honorary Research Fellow, London School of Hygiene and Tropical Medicine<br>Jenny Scott, Senior Lecturer, Bristol Medical School<br>Marisha Wickremsinhe, Research Fellow, London School of Hygiene and Tropical Medicine
**Owner(s)**	James Norman, Inclusion Health Consultant<br>Mike Brown, Consultant Infectious Diseases
**Review By Date**	15th November 2025
**Responsible Director**	Charles House, Medical Director, Medicine Board
**Monitoring Committee**	Opioid Stewardship Committee
**Target Audience**	All staff involved in caring for people who are opioid-dependent and are admitted to hospital
**Related Trust Documents / Policies**	Alcohol withdrawal guideline<br>Pathway to home (UCLH@Home)
**Keywords**	methadone; buprenorphine; opioid substitution therapy (OST); drug use; opioid withdrawal; naloxone; Prenoxad
**Number of Pages and Appendices**	30 pages, including 5 appendices
**Equalities Impact Assessment**	Low

If reading a printed copy always check that it is the most recent approved version which can be found on the Clinical Guidelines page on the intranet.

uclh

UCLH is an NHS Foundation Trust comprising: University College Hospital (incorporating the Elizabeth Garrett Anderson Wing, Grafton Way Building, Macmillan Cancer Centre and University College Hospital at Westmoreland Street) Royal London Hospital for Integrated Medicine, Royal National ENT and Eastman Dental Hospitals, National Hospital for Neurology and Neurosurgery at Queen Square and Cleveland Street, Institute of Sport, Exercise and Health, Hospital for Tropical Diseases.

University College London Hospitals
NHS Foundation Trust

uclh

Document Control Information

To be completed by Quality & Safety Department

**Version number**	2.0
**Approved By**	Clinical Guidelines Committee
**Date Approved**	11/10/2022
**Publication Date**	11/11/2022
**Document Control Number**	CPG/095/02
**Title and document control number of document this replaces**	CPG/095/01<br>Management of drug misusers (version 01)
**Summary of significant changes**	Updated language; updated prescribing guidelines in line with best practice, evidence review, and current national guidelines; removed urine drug screen requirement; added information on methadone and buprenorphine prescribing, including management after day one; clarified medicines reconciliation process; added further information on pain management for patients on OST; added provisions for takeaway naloxone; added provisions for takeaway OST for patients admitted on a community OST prescription and discharged out-of-hours

Contents

Section		Page number
1.0	Summary	2
2.0	Equality Impact Assessment	2
3.0	Introduction	2
4.0	Objectives	3
5.0	Scope	3
6.0	Abbreviation	3
7.0	Duties and responsibilities	3
8.0	Development and evidence base	3
9.0	Consultation and stakeholder involvement	4
10.0	Guidance	5
11.0	Contact details	17
12.0	Guidance implementation	18
13.0	Review, monitoring and compliance	18
14.0	References	19
15.0	Flow chart for OST prescribing	21
16.0	MEDL guideline	22
Appendix 1		23
Appendix 2		24
Appendix 3		26
Appendix 4		27
Appendix 5		30

1.0 Summary

Opioid withdrawal in hospital settings can compromise medical management and lead to discharge against medical advice. Patients who use heroin (or other illicit opioids) or receive a community prescription for opioid substitution treatment (OST) to treat opioid dependence are at risk of experiencing withdrawal symptoms in hospital. Healthcare providers can mitigate the physical symptoms and associated risks of opioid withdrawal by ensuring rapid and equitable access to OST, usually oral methadone (full agonist) or sublingual buprenorphine (partial agonist), for patients with substance dependency. When patients who receive a community prescription for methadone or buprenorphine are admitted to hospital, their routinely administered dose should be confirmed and prescribed in hospital to ensure continuity of care. If the patient's dose cannot be confirmed, or if the patient is dependent on opioids such as heroin but not in receipt of a community prescription, methadone or buprenorphine should be prescribed at a starting dose and titrated to a therapeutic dose. Preventing opioid withdrawal in hospital improves patient care and reduces the risk of discharge against medical advice and associated morbidity and mortality.

2.0 Equality Impact Statement

The authors of this guideline have undertaken an Equality Impact Assessment (EIA) and has concluded that there is no negative impact on any of the protected equalities groups. The completed EIA form is available from the Quality and Safety Department.

3.0 Introduction

People who use drugs have a higher rate of hospital admission than the general population, often to address long-term or chronic health conditions, injuries, and bacterial infections.[1-5] Many delay seeking care, and fear of opioid withdrawal in hospital remains a critical barrier to accessing and completing treatment.[6] Opioid withdrawal can be intolerable, leading patients to self-discharge in order to collect community prescriptions for methadone or buprenorphine, or to use illicit opiates.[7,8] Patients who are not assured they will promptly receive methadone or buprenorphine to prevent opioid withdrawal may self-discharge prior to developing clinically recognisable withdrawal symptoms due to psychological distress in anticipation of symptom onset.[6]

Premature discharge often leads to readmission,[9-11] which is associated with higher all-cause mortality.[12,13] Patients who self-discharge and are later readmitted may require more complex and resource-intensive care.

Opioid withdrawal is a preventable condition, and timely OST provision in hospital prevents adverse outcomes to both the individual and the healthcare system. Adequate OST provision promotes the therapeutic alliance between patients and hospital staff, thereby supporting clinicians to provide appropriate care.

Inappropriate OST provision has been associated with toxicity, and there is evidence of increased risk of all-cause mortality in the first four weeks of methadone titration as compared with the remainder of time spent on a methadone prescription in community settings.[14] These risks should be balanced against the risks of delayed or inadequate OST provision in hospital, which can lead to incomplete treatment, readmission, and overdose after discharge due to reduced tolerance.[6-8,15] Prescribing OST where clinically indicated can prevent avoidable deaths.[16]

4.0 Objectives

This guideline aims to ensure that patients with opioid dependence receive equitable hospital care by:

- Preventing or promptly treating opioid withdrawal in hospital
- Ensuring continuity between community and hospital OST

In the hospital setting, patients are generally prescribed methadone or buprenorphine to prevent and treat opioid withdrawal symptoms, either to manage an illicit opioid dependency (such as heroin) or as a continuation of their existing OST prescription.

In the community, OST generally entails the prescription of oral formulations of methadone (full opioid agonist) or buprenorphine (partial opioid agonist). See **Appendix 1** for further detail on clinical considerations specific to managing withdrawal in the hospital setting.

5.0 Scope

These guidelines are intended for all clinical staff who are involved in caring for people who use opioids, whether illicit opioids (e.g. heroin) or opioid substitution therapy, to manage opioid dependence. These guidelines apply to admitted patients who are opioid-dependent.

6.0 Abbreviations

CDTS – Community drug treatment service

COWS – Clinical Opioid Withdrawal Scale (see **Appendix 3**)

GCS – Glasgow Coma Scale

NEWS – National Early Warning Score

OST – Opioid substitution therapy, usually either oral methadone solution (1mg/ml) or sublingual buprenorphine tablets

7.0 Duties and responsibilities

Medical staff are responsible for:

- Taking an appropriate drug history
- Performing the physical examination
- Prescribing STAT OST dose (day one)
- Prescribing PRN OST (day one), if indicated
- Prescribing PRN naloxone
- Prescribing daily dose (day two onward)
- Contacting community prescriber and/or pharmacist to confirm dose and last consumption (where relevant) for patients admitted on a community prescription
- Informing the UCLH Drug and Alcohol Liaison CNS via EPIC referral of all admissions requiring OST prescription
- Liaising with community drug treatment services as part of discharge planning

- Prescribing takeaway naloxone on discharge, and where necessary and indicated, take-home OST in line with this guideline

Nursing staff are responsible for:

- Informing the UCLH Drug and Alcohol Liaison CNS via EPIC referral of all admissions requiring OST prescription
- Monitoring symptoms of opioid withdrawal and offering PRN doses of OST as prescribed, if needed
- Monitoring symptoms of opioid toxicity and administering naloxone if indicated
- Liaising with community drug treatment services as part of discharge planning
- Training patients in the use of takeaway naloxone and providing a sharps bin

Ward pharmacists are responsible for:

- Ensuring that OST is correctly prescribed
- Contacting community prescriber and/or pharmacist to confirm dose and last consumption (where relevant) for patients admitted on a community prescription
- Liaising with community drug treatment services as part of discharge planning

8.0 Development and evidence base

This guideline was developed as part of the NIHR-funded Improving Hospital Opioid Substitution Therapy study (NIHR133022, PI: M. Harris). The guideline was written in consultation with people who use opioids as well as clinicians, nurses, and pharmacists working in acute hospital settings.

The evidence base to support the development of this guideline draw from multiple sources. [A full bibliography is included in Section 14.0]. Key references include:

- British National Formulary (BNF) – Methadone and buprenorphine monographs
- NICE Guidance on methadone and buprenorphine for the management of opioid dependence (TA114)
- Department of Health and Social Care. *Drug misuse and dependence UK guidelines on clinical management* ('the Orange Book')
- Faculty of Pain Medicine recommendations on acute pain management for patients on OST

The development of this guideline was also informed by a robust policy analysis of 101 policies related to opioid withdrawal management across 86 trusts in England by Harris et al.[17] Harris et al. characterized current practice for OST provision in acute hospital settings with respect to a number of critical aspects of OST provision, including assessment procedures, protocols for continuation of OST for patients on a community prescription, protocols for initiating and titrating OST for patients not on a community prescription or whose community prescription cannot be confirmed, pain management for patients receiving OST, and discharge procedures.[17] Development of this guideline was also underpinned by evidence generated through the 'Care and Prevent' study[18] (PI: M. Harris) which identified delayed or omitted OST as a primary barrier to hospital attendance by people who use opioids, and as a key cause of discharge against medical advice for people who use opioids.[6]

9.0 Consultation and stakeholder input

The guideline was initially drafted by Dr Magdalena Harris, Dr Adam Holland, Dr Jenny Scott, and Dr Marisha Wickremsinhe in consultation with the iHOST Policy Template Working Group (Dr Tommy Brothers; Dr Mike Brown; Niamh Eastwood, Roz Gittins, Dr Chris Hallam, Prof Viv Hope, Mariebeth Kilbride, Dr Dan Lewer, Claire Robbins, Fenella Sentence, Katherine Watkinson, Dr Tom Yates).

The iHOST Policy Oversight Group reviewed and contributed to the development of this guideline:

- Dr Steve Brinksman, Clinical Director Addiction Professionals and Medical Director Cranstoun
- Dr Nicky Kalk, Consultant Addictions Psychiatrist, King's College London
- Dr Andrew Scourfield, Consultant in Clinical Pharmacology and General Internal Medicine, University College London Hospitals NHS Foundation Trust
- Chris Todd, MPharm, MCHMP, Pharmacist, College of Mental Health Pharmacy
- Laura Wilson, MRPharmS. Practice and Policy Lead – Scotland, Royal Pharmaceutical Society

The guideline was also revised following feedback from:

- Dr Mike Kelleher – Office for Health Improvements and Disparities
- Laura Pechey – Office for Health Improvements and Disparities
- Steve Taylor – Office for Health Improvements and Disparities
- Prof Sir John Strang - National Addiction Centre, Institute of Psychiatry, Psychology & Neuroscience (IoPPN), King's College London
- Adrian 'Bean' Noctor, Outreach Specialist, Find and Treat Care Team (UCLH)
- Dr James Norman, Inclusion Health Consultant (UCLH)
- Kate Robinson, Drug and Alcohol Liaison CNS (UCLH)
- UCLH Opioid Stewardship Committee
- UCLH Use of Medicines Committee
- UCLH Clinical Guidelines Committee

10.0 Guidance

10.1 Key principles of OST provision in hospital

- NICE recommends OST to manage opioid dependence.[19] OST reduces the risk of all-cause mortality, including from opioid overdose, and reduces the risk of blood-borne virus transmission, including HIV and hepatitis C.[20]

- In hospital, the purpose of OST provision is to:
  1) Prevent opioid withdrawal symptoms,
  2) Enable treatment of presenting medical condition(s),
  3) Reduce risk of self-management of withdrawal using illicit opioids in hospital, and
  4) Reduce risk of discharge against medical advice.

- While excess OST can lead to toxicity, inadequate OST provision poses significant risks, including reduced opioid tolerance and discharge against medical advice, which can increase the likelihood of fatal overdose on discharge.[15] Insufficient OST provision can also lead to inpatient use of illicit opioids. These risks can be managed through adequate OST provision in hospital and comprehensive discharge planning, including provision of takeaway naloxone (the antidote to opioid overdose) where appropriate.

- Where the patient is not in receipt of a community OST prescription, or their opioid tolerance is unknown, careful observation and titration of OST allows for appropriate management of opioid withdrawal symptoms.

- An established maintenance dose of OST does not offer analgesic effect to treat acute pain in patients with opioid dependence. Medication for pain management must be prescribed in addition to OST.

- Key wards where patients who require OST prescription are commonly managed will need to have OST supplies in stock, with arrangements made to provide timely access to OST (methadone or buprenorphine) where patients are transferred to other wards.

10.2 Patient assessment

Prompt assessment and management of opioid withdrawal, with clear communication throughout, can promote a collaborative relationship between patient and clinician and enable the successful management of other medical conditions. On admission, patients should be informed that any symptoms of opioid withdrawal will be managed promptly and appropriately. Patients should be assured that there is no need to acquire community OST prescriptions or illicit opiates whilst in hospital.

To assess the need for OST in hospital, a comprehensive patient history and physical examination should be conducted.

<u>10.2.1. Patient history</u>

- Opioid use
  - Community OST prescription:
    - Medication
    - Formulation*
    - Dose
    - Frequency of pick-up
    - If the community OST prescription is supervised
    - Whether the patient has brought doses with them, and if so, the quantity brought in
  - Other opioid use (including in addition to prescribed OST):
    - Amount
    - Frequency
    - Route of administration
    - Duration of use
  - Date and time since last opioid dose (onset of withdrawal symptoms is approximately 4 hours after last heroin dose or 24 hours after last methadone dose)
- Experience of withdrawal symptoms
  - Current experience of withdrawal symptoms, if any
  - Past experiences of withdrawal symptoms
- Concurrent drug use or polysubstance use
  - Amount, frequency, route, duration of use

*Some patients may be admitted on injectable OST (e.g., diamorphine) or depot OST (e.g. Buvidal) to manage opioid dependence. These patients must be discussed with community drug treatment services and the Alcohol and Drug Liaison CNS at the earliest opportunity plans for continuity of treatment. These patients should *not* be prescribed methadone or buprenorphine according to this guideline.

- Current experience of withdrawal symptoms, if any

NB: Severe alcohol withdrawal is a medical emergency and should be promptly managed per local guidelines [LINK].

Opioid withdrawal symptoms may mask alcohol or benzodiazepine withdrawal symptoms. Caution is advised when managing opioid withdrawal alongside alcohol or benzodiazepine withdrawal considering the risks of additive sedation. Both withdrawals should be treated simultaneously, and the patient's respiratory rate, oxygen saturation, and GCS should be closely monitored. More cautious OST titration may be necessary.

See **Appendix 2** for further considerations when taking a comprehensive drug history.

10.2.2. Physical examination

- Signs and symptoms of opioid withdrawal (consider assessment using validated scoring tool such as the Clinical Opioid Withdrawal Scale (COWS), **Appendix 3**)

Early signs and symptoms	Restlessness, anxiety, agitation, discomfort, yawning
Intermediate signs and symptoms	Sweating, runny nose, teary eyes, hot / cold flushes, dilated pupils, irritability, loss of appetite
Late signs and symptoms	Muscle spasms, insomnia, abdominal cramps, low-grade fever, nausea and vomiting, tachycardia, hypertension, diarrhoea, tremors, goosebumps, bone and muscle aches

- Signs of opioid toxicity

Moderate toxicity	Difficulty keeping eyes open (sedation), drowsiness, shallow breathing, constricted pupils
Severe toxicity	Coma, reduced respiratory rate, bradycardia, hypotension, hypothermia

NB: All patients who receive OST in hospital should be routinely monitored for signs of opioid toxicity four hours after every OST dose is given and then as per NEWS.

- Signs of health conditions associated with injecting drug use that may require treatment (e.g., abscesses and other skin and soft tissue infections, venous thromboembolism, subacute bacterial endocarditis)

A comprehensive patient history to determine opioid dependence and a physical examination to identify presence of withdrawal symptoms should be sufficient to indicate OST provision. Supporting evidence of opioid dependence may be found in previous hospital records, discharge summaries, clinic letters, and medication charts.

For patients who are on a confirmed community prescription or for patients whose clinical history, presentation, or other supporting evidence is consistent with opioid dependency, drug tests such

as urine drug screens are unlikely to offer any additional confirmation or clinical indication of whether OST is needed. Drug screens are designed to confirm recent opioid use, not to prove opioid dependence, and overreliance on testing combined with the possibility of false negative results[21] may compromise timely and appropriate OST provision. Moreover, patients may experience drug screens as stigmatizing.

Where a drug screen is requested, either to enable further targeted discussion with the patient about their drug use or in cases of considerable uncertainty regarding the patient's current opioid use, care should be taken to explain to the patient why testing is needed. The clinician should make clear that an appropriate and timely OST dose will be provided and that treatment of withdrawal symptoms will not be delayed by waiting for the test results. Drug tests should only be pursued with patient consent.

NB: Where withdrawal symptoms are clearly present, the patient's decision to withhold a test sample should not preclude initiation of OST.

10.2.3. Confirming community OST prescription

A patient's community OST prescription should be promptly continued in hospital. Delays in OST provision increase the likelihood of intolerable withdrawal symptoms and discharge against medical advice.

Ask the patient to verify the details of their OST prescription:
- Medication,
- Formulation,
- Daily dose,
- Prescription and pick-up frequency, including whether consumption is supervised or unsupervised,
- Usual time of consumption, and
- Date and time of the last dose taken.

If assessing the patient during working hours, contact the patient's community pharmacist or the patient's OST community prescriber (drug treatment service, GP) to confirm medication, formulation, dose, and whether consumption is supervised. If consumption is supervised, contact the patient's community pharmacist to confirm date of last consumption. Note that many community pharmacies are open on weekday evenings and on the weekends.

For patients who are on an unsupervised prescription (e.g., some patients receive weekly or twice weekly takeaway doses), confirm date and time of last consumption with the patient themself. Patients who are on unsupervised prescriptions tend to be considered lower risk as they are well established on their OST. This may not be the case during public health emergencies, such as the COVID-19 pandemic, when many clients were given take-home (i.e., unsupervised) OST doses out of necessity.

Inform both the community prescriber and the community pharmacist of the date of the patient's hospital admission and ensure that the patient's usual community prescription is put on hold.

All communication with the community drug treatment service and community pharmacy should be clearly documented in the patient's medical records.

If the prescriber or pharmacist are unavailable (i.e., if the patient presents out-of-hours), alternative sources of dose confirmation may include:

- Key worker (some services have an out-of-hours contact number)
- Labelled and recently issued OST medication bottles/prescriptions brought to hospital by patient

The following sources may be used to confirm opioid dependence but not necessarily dose:

- Recent previous hospital notes, discharge summaries, and drug charts (note that routinely prescribed doses of OST may have changed since last admission, or the patient may have lost tolerance to that dose since last admission)
- Summary Care Records (SCR) or Health Information Exchange (HIE) (note that doses of OST may frequently change and drug treatment services do not have access to summary care records to input or update dosing information)

10.2.4. Prescribing naloxone as required

All patients requiring OST in hospital, including patients on a community OST prescription, should be prescribed naloxone as required on Epic to treat any severe toxicity from OST or illicit opioid use during their hospital admission.

How to administer naloxone in case of overdose:

Naloxone should be administered intravenously at an initial dose of 400 micrograms. If no response, two doses at 800 micrograms each can be repeated at one-minute intervals. If still no response, increase to 2mg dose for one dose. Patients with severe poisoning may require 4mg dose. Review diagnosis; further doses may be required if respiratory function deteriorates.

Naloxone has a shorter half-life than methadone and other opiates, meaning the patient may experience recurring symptoms of severe toxicity after naloxone wears off. The patient should be closely monitored for signs of recurrent overdose for 24 hours, as multiple administrations may be needed. Note that naloxone can precipitate severe opiate withdrawal.

10.3. Continuing a community OST prescription in hospital

Use the Epic SmartPhrase **.methadoneOST** or **.buprenorphineOST** prior to prescribing inpatient OST so that all relevant information is documented.

See BNF guidelines for additional information on methadone and buprenorphine prescribing.

NB: Methadone and buprenorphine are contraindicated and should not be prescribed if the patient has acute respiratory depression, head injury, raised intracranial pressure, risk of paralytic ileus or is comatose. Methadone is also contraindicated for patients with phaeochromocytoma.

Split (i.e., twice-daily) dosing is advised where there are cautions such as risk factors for QT-interval prolongation, impaired respiratory function from chronic obstructive pulmonary disease (COPD) or asthma, decompensated liver disease, renal impairment or concomitant use of other CNS depressants (e.g., benzodiazepines) or gabapentinoids. Where relevant, split dosing is also advised to facilitate pain management (see Section 10.5).

For all patients admitted on a community prescription, ensure good communication with the community drug treatment service to inform them of the patient’s admission, ensure speedy medicines reconciliation, and make arrangements for discharge planning (including suitability for bridging prescription if discharged out-of-hours). Document all communication with the community drug treatment service in the patient’s notes.

10.3.1. For patients with a community prescription for methadone

Use the Epic SmartPhrase **.methadoneOST** prior to prescribing inpatient methadone OST so that all relevant information is documented.

Where the dose is confirmed by the community prescriber or pharmacist, where the patient has taken their prescribed dose within the three days prior to hospital admission, and where there are no contraindications or cautions, the patient’s full dose of methadone should be prescribed and administered once daily, as in the community setting, ideally in the morning.

Ensure good communication with the patient; some patients, especially those on unsupervised consumption, may prefer split dosing. Highlight the potential risk of overdose if the patient receives their full prescribed dose in hospital but has not been taking their full dose regularly at home.

If a patient is on a community prescription for methadone but has not taken their usual dose within three days of admission (confirmed by the pharmacist if supervised or by the patient if unsupervised), their tolerance may be reduced. In this case, OST should be prescribed according to a titration schedule (see Section 10.4.1).

If a patient is on a community prescription for methadone but the dose cannot be confirmed (see Section 10.2.3), OST should be prescribed according to a titration schedule (see Section 10.4.1) until the community dose (and date of last consumption, if supervised) is confirmed by the community pharmacist and/or prescriber.

On the day the dose is confirmed, and where the last consumption was within three days of the date of dose confirmation, the patient should receive an additional dose of methadone to meet the total daily dose of their community prescription, if needed. The full dose should be prescribed from the next day.

Some patients receiving methadone in the community use additional opioids (e.g., heroin) on top of their prescribed OST. In these cases, patients may experience withdrawal symptoms despite taking their full prescribed dose. Patients on a community prescription of methadone should be monitored for symptoms of withdrawal and prescribed an additional 5-10mg of PRN methadone (maximum 10mg per day) if opioid withdrawal symptoms (see Section 10.2.2.) are still present

four hours after administration of their full community dose. The goal of PRN doses is to ensure that patients maintain their tolerance, thereby reducing risk of overdose at discharge. If the patient requires additional methadone dose(s) to prevent withdrawal symptoms, their once daily dose should be increased to account for PRN doses, not exceeding an increase of more than 30mg over seven days.

Community drug treatment services should be informed of any additional doses of methadone given in hospital that might necessitate changes to their community prescription after discharge.

10.3.2. For patients with a community prescription for buprenorphine

Use the Epic SmartPhrase **.buprenorphineOST** prior to prescribing inpatient buprenorphine OST so that all relevant information is documented.

Where the dose is confirmed by the community prescriber or pharmacist, where last consumption (as reported by the pharmacist if supervised or the patient if unsupervised) was less than three days prior to admission, and where there are no contraindications or cautions, the patient’s full dose of buprenorphine should be prescribed and administered once daily, as in the community setting. Buprenorphine should be prescribed according to a titration schedule (see Section 10.4.2) where the last dose was consumed more than three days prior to hospital admission.

If a patient is on a community prescription for buprenorphine but the dose (and last consumption, if supervised) cannot be confirmed (e.g., out-of-hours), OST should be prescribed according to a titration schedule (see Section 10.4.2).

As a partial agonist, buprenorphine minimizes the euphoric effects of an additional or on-top opioid use. By nature of its activity, patients who regularly take buprenorphine are unlikely to be using additional opioids on top of their OST, and therefore are unlikely to require PRN buprenorphine to maintain tolerance.

10.4. Initiating or re-titrating OST in hospital

Use the Epic SmartPhrase **.methadoneOST** or **.buprenorphineOST** prior to prescribing inpatient OST so that all relevant information is documented.

If a patient who is not already on a community prescription is assessed to be opioid-dependent with features of withdrawal or at risk of developing withdrawal symptoms, OST should be initiated in hospital. For patients who are on a community prescription but whose community dose cannot be confirmed, or whose last dose was consumed more than three days before admission, OST (using their routine medication) should be re-titrated in hospital.

An optimal dose of OST prevents withdrawal symptoms for at least 24 hours. Where a patient’s tolerance is unknown, titration to promptly manage withdrawal symptoms is critical to ensure that the patient remains comfortable in hospital and does not need to acquire opioids from outside the hospital to treat their withdrawal symptoms., Where routine monitoring is available, the hospital environment can support a higher initiation dose of OST than might otherwise be considered in the community.

Patients who do not receive OST in the community can be initiated on methadone or buprenorphine. The choice of medication depends on patient preference, medicine availability, and relevant clinical considerations (see **Appendix 1** for further detail on clinical considerations specific to managing withdrawal in the hospital setting). The management plan should be discussed and agreed with the patient. The dose of OST should then be titrated against the patient's withdrawal symptoms, as described below.

If the patient refuses OST medications for withdrawal symptoms, offer symptomatic management. Explain to the patient that OST is recommended to treat opioid withdrawal and that OST prescription is therefore advised.

Even where a patient has consented to receiving OST, consider offering adjuvant medications as required to address any severe symptoms of withdrawal (e.g., antiemetics to enable methadone consumption).

For all patients initiated on OST in hospital, inform the local drug treatment service of the admission to facilitate a referral pathway on discharge.

10.4.1. Initiating or re-titrating methadone

Use the Epic SmartPhrase **.methadoneOST** prior to prescribing inpatient methadone OST so that all relevant information is documented.

Day one prescribing
For a patient showing signs of opioid withdrawal (see Section 10.2.2) who is not on a community prescription, whose community prescription cannot be confirmed, or whose last dose was consumed more than three days prior to hospital admission, a starting dose of 20mg of methadone should be prescribed by medical staff. This dose should be given as soon as possible assuming withdrawal symptoms are present, and within 24 hours of the patient's last reported dose of methadone, where relevant. Where the patient's clinical history suggests high opioid tolerance, and where regular monitoring is available, a starting dose of 30mg may be prescribed.

If there are medical cautions (see Appendix 1) for methadone, the starting dose can be split: 10 mg should be prescribed in the first instance, and the patient should be monitored for ongoing withdrawal symptoms. The patient should also be monitored for signs of opioid toxicity, e.g., sedation, respiratory depression (see Section 10.2.2). After four hours, the remaining 10mg of the initial dose should be administered by nursing staff.

Nursing staff should reassess the patient for opioid withdrawal symptoms (e.g., using COWS) four-hourly from initial dosing (the time taken for methadone to reach peak concentrations). If the patient is still showing signs of withdrawal after four hours (e.g., COWS score greater than or equal to 5), an additional 10mg PRN methadone should be administered by nursing staff. The patient should again be reassessed for opioid withdrawal symptoms after four hours (see Section 10.2.2); if symptoms of withdrawal are still present, an additional 10 mg PRN methadone should be administered by nursing staff. The patient should be closely monitored for continuing

withdrawal symptoms. During initiation or re-titration, the upper bound of the day-one dose of methadone is normally 40mg.

- If the patient still shows signs of withdrawal four hours after a total dose of 40mg is given, and if there are no contraindications or clinical concerns, an increased day-one dose of up to a maximum 60mg may be appropriate, prescribed and administered in 10mg increments four-hourly. The decision to prescribe more than 40mg on day one must be made under expert supervision, e.g., Drug & Alcohol CNS, specialist pharmacist, inclusion health consultant, or a senior resident medical, or anaesthetic clinician. During initiation or re-titration, more than 40mg of methadone should only be prescribed and administered on day one if the patient is on a ward where direct, routine observations are possible, and patients should be closely monitored for symptoms of opioid toxicity four hours after every OST dose given and then as per NEWS (see Section 10.2.2).

Patients who are administered a high dose of methadone on day one must be carefully monitored for opioid toxicity for several days. Methadone has a long and variable half-life (between 13 and 47 hours with repeated doses[22]), with wide inter-individual variability in methadone metabolism across patients. Even where the daily dose remains constant, the patient's methadone plasma levels can continue to accumulate up to ten days[21] (or longer if renal or hepatic impairment), resulting in delayed toxicity and possible overdose.

If the patient's respiratory rate is less than 12, if oxygen saturation is below target, or if the patient has a reduced level of consciousness, any scheduled OST dose should be withheld and the ward doctor should be called to review future doses. If signs of overdose, naloxone should be administered (see Section 1.4).

Day two and onward prescribing
Assuming no over-sedation on day-one, on day two, the total day-one dose should be split into a twice daily prescription and prescribed by medical staff. Twice daily dosing is preferred until the patient's dose is stabilised given the potential risk of overdose during initiation due to methadone's cumulative effect.

The patient should continue to be monitored for symptoms of opioid withdrawal (see Section 10.2.2).

For patients prescribed more than 40mg on day one, the dose should only be increased, if required, every second day by no more than 10mg per day.

For patients prescribed 40mg or less on day one, the dose can be increased by no greater than 10mg methadone per day if the patient is still experiencing withdrawal symptoms on day two.

If the patient's daily dose is increased on day two to treat continued withdrawal symptoms, prescribe the total day-two dose (i.e., day-one dose plus additional PRN methadone up to 10mg) as a split (i.e. twice-daily) dose on day three. If withdrawal symptoms persist after four hours of giving the total dose on day three, another 10mg PRN methadone dose increase can be prescribed.

The maximum weekly increase in methadone to treat persisting withdrawal symptoms should not exceed 30mg above the day-one dose (i.e. after seven days, the daily dose should not exceed the day one dose by more than 30mg).[23] For example, if 40mg was prescribed on day one, the daily dose on day seven should not exceed 70mg. Continue to monitor for signs of opioid toxicity four-hourly (see Section 1.2).

NB: Given methadone's cumulative effect, patients must be carefully monitored throughout their hospital stay, even where the dose has not been increased. If the patient shows signs of toxicity, the daily methadone dose should be reviewed and considered for dose reduction.

From the second week onward, any required dose increases (of no more than 10mg on any one day) should be administered a few days apart as blood levels will have accumulated by this time. Assuming no withdrawal symptoms are present, further dose increases are not recommended.[21]

Once the patient has been stabilised on their dose, convert the prescription to a once-daily dose. Stabilisation occurs when there are no objective signs of opioid withdrawal (e.g., per COWS), when there are no subjective signs of opioid withdrawal per the patient's self-report, and when there are no signs over opioid toxicity (i.e. over-sedation).

In the community, stabilised methadone doses range from 60-120mg,[19,23] but doses on both sides of this range are routinely used in practice, guided by response.

10.4.2. Initiating or re-titrating buprenorphine

Use the Epic SmartPhrase **.buprenorphineOST** prior to prescribing inpatient buprenorphine OST so that all relevant information is documented.

The following dosing recommendations are for sublingual buprenorphine initiation as OST.[21]

Given its lower risk of respiratory depression (as compared with methadone), buprenorphine may be preferred where tolerance is uncertain in patients who are not already on a community prescription.

NB: Buprenorphine should only be administered if symptoms of withdrawal are present. As a partial opioid agonist, buprenorphine can precipitate opioid withdrawal in patients who have recently taken a full opioid agonist like heroin or methadone.

Many patients are familiar with buprenorphine and know when a dose of buprenorphine should be administered without precipitating withdrawal. Confirm that the administration time of the initial dose of buprenorphine is acceptable to the patient (i.e., that buprenorphine will not precipitate withdrawal symptoms).

Day one prescribing
Patients experiencing symptoms of opioid withdrawal should be prescribed 4mg of buprenorphine (sublingual) and monitored for signs of opioid toxicity (see Section 10.2.2). To prevent

precipitated withdrawal, only administer buprenorphine when symptoms of withdrawal are present.

If the patient is still experiencing withdrawal symptoms after four hours, an additional 2 mg PRN buprenorphine can be administered, up to a maximum total dose of 8mg on day one.

Day two and onward prescribing
On day two, the total day-one dose should be converted into a once-daily prescription. The patient should continue to be routinely monitored for withdrawal symptoms; if still experiencing withdrawal symptoms, the patient should be prescribed and administered an additional 4mg PRN buprenorphine four-hourly up to a maximum total dose of 16mg on day two. On day three, convert the total day-two dose into a once-daily prescription and continue to titrate against withdrawal symptoms up to a maximum of 32 mg per day.

In the community, usual doses of buprenorphine tend to range from 12-24mg, up to a maximum of 32mg for sublingual formulations.

10.5. Acute pain management for patients on methadone or buprenorphine

Maintenance OST doses do not provide analgesic effect for acute pain. Patients on OST with high opioid tolerance may also have increased pain sensitivity, requiring larger doses of opioid analgesia.[24] The following recommendations are drawn from the Faculty of Pain Medicine guidelines on acute pain management for people who receive OST.

10.5.1. For patients taking methadone

- Split routine dose and administer BD or TDS (to allow for monitoring of opioid toxicity symptoms)
- Titrate additional opioid analgesia against acute pain symptoms

10.5.2. For patients taking buprenorphine

- Split routine dose and administer BD or TDS (to allow for monitoring of opioid toxicity symptoms)
- Titrate additional opioid analgesia against acute pain symptoms

NB: Buprenorphine is a partial opioid antagonist, so may reduce the effectiveness of opioid analgesics. If analgesia is insufficient to manage acute pain symptoms, additional non-opioid analgesics can be used, or buprenorphine can be discontinued and switched to methadone to optimise pain management. *See clinical guidelines for further advice on switching from buprenorphine to methadone.*

For further advice on pain management for patients on methadone or buprenorphine, see Faculty of Pain Medicine guidelines. Consider referral to the UCLH pain team.

10.6. Discharge

Use the Epic SmartPhrase **.OSTdischarge** to document the discharge actions required for safe discharge of patients on OST.

For all patients:
On the day of discharge, the patient's full methadone or buprenorphine dose should be administered in hospital.

Considering the elevated risk of fatal overdose in the two days immediately following hospital discharge[15], the patient should also be offered naloxone for take-home use (2mg/2mL pre-filled syringe) to reduce the risk of fatal overdose in the community post-discharge. Where possible, takeaway naloxone should also be offered to family members and friends of the patient.

Nursing staff should train patients in the use of naloxone to prevent fatal opioid overdose (see **Appendix 4** for relevant resources, including a training checklist):

- Ensure that the patient and/or family or friend knows how to recognize the signs of overdose, how to administer Prenoxad®, and knows to call 999 in case of overdose.
- Emphasize the importance of carrying naloxone on their person.
- Explain to the patient that their tolerance may have changed during their hospital stay and encourage them to take additional precautions with opioid use or other substance use following discharge
- Encourage the patient not to use drugs alone and to ensure that the people they are with when using have access to naloxone and know how to administer it.

10.6.1. For patients on a community prescription for methadone or buprenorphine

Use the Epic SmartPhrase **.OSTdischarge** to document the discharge actions required for safe discharge of patients on OST.

Advanced discharge planning is essential to ensuring continuity of care following hospital discharge. Hospital discharge can represent a 'pinch point' for transferring patients back into the care of community drug treatment services. To enable continuity of OST prescription, hospital staff should prioritise discharge planning as much as possible.

Discharge planning should include clear communication with the patient's community prescriber and community pharmacist to ensure that the patient's community prescription is continued from the day after discharge. Community providers should also be informed of any changes to the methadone or buprenorphine dose prescribed in hospital. Ideally, patients should be discharged in-hours to ensure seamless transfer of care from the hospital to the patient's community drug treatment service.

Good discharge planning and clear communication with the patient's community drug treatment service is essential to enable continuity of care and prevent a break in OST access. Where the patient cannot be discharged in-hours, ensure that arrangements have been made to continue the prescription in the community from the day after discharge.

Where the patient is discharged out-of-hours (i.e., weekends, bank holidays) but the community drug treatment service is not available to restart the patient's prescription from the day after discharge, takeaway dose(s) of methadone or buprenorphine can be prescribed. The aim of takeaway OST is to ensure continuity of care until OST can be provided in the community.

Where agreed with the community drug treatment service or community prescriber, OST can be supplied on a takeaway basis in line with UCLH's standard processes. Takeaway OST should be prescribed for as short a time period as possible (usually one to three days) as a strategy for bridging the patient from hospital care back into community care. Methadone should be dispensed with an appropriate measuring cup.

For example, if a patient is discharged on a Friday afternoon, and the community prescriber is not available to restart the patient's prescription until Monday, the patient should receive their once-daily dose in hospital on Friday morning before discharge, and should be given takeaway doses for Saturday, Sunday, and Monday (to ensure the patient is well enough to travel to the drug treatment service or GP on Monday).

For patients who are being discharged with a new referral to a nursing or care home, additional takeaway OST may be needed to ensure continuity of care. In these cases, up to 7 days of takeaway OST can be prescribed in consultation with community drug treatment services and the Drug and Alcohol Liaison CNS.

NB: Takeaway OST should only be prescribed where discussion with the community drug treatment service (or community prescriber) confirming the suitability of takeaway OST for the particular patient, in light of both the patient's circumstances and the nature of the admission, has taken place and the community drug treatment service (or community prescriber) has agreed that the patient can be offered takeaway OST if discharged out-of-hours.

Ensure that the community prescriber and community pharmacist are informed of all takeaway OST dose(s).

Takeaway OST should only be considered where the community drug treatment service is not able to guarantee routine OST prescribing from the day following discharge. **Takeaway OST is not authorised where agreement with the community drug service has not been obtained.**

10.6.2. For patients initiated on methadone or buprenorphine in hospital

Use the Epic SmartPhrase **.OSTdischarge** to document the discharge actions required for safe discharge of patients on OST.

Recognising that hospital discharge can be a high-risk period for patients who use opioids, an urgent appointment must be arranged with the community drug treatment service, scheduled for the day of discharge, to enable continuity of care from hospital OST to community OST for all patients initiated on methadone or buprenorphine in hospital.

If a same-day appointment cannot be made on the day of discharge, a bridging prescription may be arranged with the community drug treatment service, where possible, to ensure continuous

access to OST until the patient can be seen in the community. If the community drug treatment service agrees to arrange a bridging prescription for the patient, ensure that a copy of the discharge summary, as well as confirmation of inpatient prescribing, is shared with the community drug treatment service team. **Methadone or buprenorphine should not be supplied on the discharge prescription for newly initiated patients.**

11.0 Contact details

- The Margarete Centre (Camden Drug Service): 108 Hampstead Rd, NW1 2LS Tel: 020 3317 6000
- Better Lives Islington
  - Drug services: 99-101 Seven Sisters Rd, Finsbury Park, London N7 7QP Tel: 0203 317 6099
  - Alcohol services**:** 309 Gray's Inn Road, WC1X 8QS
- Integrated Camden Alcohol Treatment Service (ICATS): Tel 020 3227 4950
- Islington Specialist Alcohol Treatment Service (ISATS): Tel: 020 3317 6650
- Camden Health Improvement Pathway (CHIP): 108 Hampstead Road, NW1 Tel: 0203 182 4200
- The Hickey Practice: Cardinal Hulme Centre 3 Arneway Street SW1P 2BG. Tel: 0207 222 8593
- WDP INROADS (Intensive Recovery Outreach and Dedicated Support); Tel: 0300 303 4545
  - INROADS is a street support service for rough sleepers and those at risk of returning back to the street (age 18+) who are experiencing substance misuse issues in Camden and Islington; INROADS works to support rough sleepers and people in temporary accommodation back into mainstream drug and alcohol treatment services by offering assessment, rapid prescribing, and engagement into community care services
- London MECC (lists community drug treatment services across London): https://www.mecclink.co.uk/london/alcohol-drugs/

Contact details for the patient's community pharmacy may be recorded in the patient's Summary Care Record (SCR) or Health Information Exchange (HIE). If the patient arrives to hospital with a 'MyMeds' card, the contact details of their local pharmacy will be listed.

12.0 Guidance implementation

- Publication on intranet (including MEDL guideline)
- Trainings for staff:
  - Visits to departmental governance and educational meetings to highlight new guidance and answer questions with local designated medical pharmacy and local nursing leads
  - E-learning training component developed through iHOST study [LINK to be added]
- Policy change 'launch' to be announced through UCLH communications newsletter
- Incorporation of prescribing recommendations into Epic order set(s)

13.0 Review, monitoring and compliance

**1. Key process/part of this policy for which compliance or effectiveness is being monitored**	**2. Monitoring method (i.e. audit, report, on-going committee review, survey etc.)**	**3. Job title and department of person responsible for leading the monitoring**	**4. Frequency of the monitoring activity**	**5. Monitoring Committee responsible for receiving the monitoring report/audit results etc.**	**6. Committee responsible for ensuring that action plans are completed**

• Time to OST prescription<br>• Administration delays and dose omissions of prescribed OST<br>• Administration (rather than prescription) of naloxone in hospital (i.e., OST-related adverse events)<br>• Number of patients prescribed OST who discharge against medical advice<br>• Number of patients prescribed takeaway naloxone	• Audit report on adverse events<br>• DATIX reports<br>• Administration of naloxone related to overdose<br>• Case review of patients with opioid dependence who discharge against medical advice	James Norman, Inclusion Health Consultant	Quarterly	Local governance committees (e.g. AMU clinical governance, T8 clinical governance)	Opioid Stewardship Committee

14.0 References

1. Lewer D, Tweed EJ, Aldridge RW, Morley KI. Causes of hospital admission and mortality among 6683 people who use heroin: A cohort study comparing relative and absolute risks. *Drug Alcohol Depend*. 2019;204:107525;
2. Wright T, Hope V, Ciccarone D, et al. Prevalence and severity of abscesses and cellulitis, and their associations with other health outcomes, in a community-based study of people who inject drugs in London, UK. *PLoS One*. 2020 ;15(7)
3. Doran J, Harris M, Hope V, et al. Factors associated with skin and soft tissue infections among people who inject drugs in the United Kingdom: a comparative examination of data from two surveys. *Drug Alcohol Depend*. 2020;213(1)
4. Lavender T, McCarron B. Acute infections in intravenous drug users. *Clin Med*. 2013;13(5):511–3
5. Lewer D, Harris M, Hope V. Opiate injection-associated skin, soft tissue, and vascular infections, England, UK, 1997-2016. *Emerg Infect Dis*. 2017;23(8):1400–3
6. Harris M. Normalised pain and severe health care delay among people who inject drugs in London: Adapting cultural safety principles to promote care. *Soc Sci Med*. 2020;260:113183
7. McNeil R, Small W, Wood E, et al. Hospitals as a 'risk environment': an ethno-epidemiological study of voluntary and involuntary discharge from hospital against medical advice among people who inject drugs. *Soc Sci Med*. 2014;105:59–66
8. Simon R, Snow R, Wakeman S. Understanding why patients with substance use disorders leave the hospital against medical advice: a qualitative study. *Subst Abus*. 2020 ;41(4) :519–25.
9. Choi M, Kim H, Qian H, et al. Readmission rates of patients discharged against medical advice: a matched cohort study. *PloS One*. 2011 ;6(9)

10. Pages K, Russo J, Wingerson D, et al. Predictors and outcome of discharge against medical advice from the psychiatric units of a general hospital. *Psychiatr Serv*. 1998;49(9):1187–92
11. Eaton E, Westfall A, McClesky B, et al. In-hospital illicit drug use and patient-directed discharge: barriers to care for patients with injection-related infections. Open forum. *Infect Dis Ther*. 2020;3(3)
12. Glasgow J, Vaughn-Sarrazin M, Kaboli P. Leaving against medical advice (AMA): risk of 30-day mortality and hospital readmission. *J Gen Intern Med*. 2010;25(9):926–9
13. Southern W, Nahvi S, Arnsten J. Increased risk of mortality and readmission among patients discharged against medical advice. *Am J Med*. 2012;125(6):594–602
14. Santo T, Clark B, Hickman M, Grebely J, Campbell G, Sordo L, Chen A, Tran LT, Bharat C, Padmanathan P, Cousins G. Association of opioid agonist treatment with all-cause mortality and specific causes of death among people with opioid dependence: a systematic review and meta-analysis. *JAMA Psychiatry*. 2021;78(9):979-93
15. Lewer D, Eastwood B, White M, Brothers TD, McCusker M, Copeland C, Farrell M, Petersen I. Fatal opioid overdoses during and shortly after hospital admissions in England: A case-crossover study. *PloS Medicine*. 2021;18(10):e1003759
16. Darke S, Larney S, Farrell M. Yes, people can die from opiate withdrawal. *Addiction*. 2017;112(2):199-200
17. Harris M, Holland A, Lewer D, Brown M, Eastwood N, Sutton G, Sansom B, Cruickshank G, Bradbury M, Guest I, Scott J. Barriers to management of opioid withdrawal in hospitals in England: a document analysis of hospital policies on the management of substance dependence. BMC Med. 2022;20(1):151.
18. Harris M, Brathwaite R, McGowan CR, Ciccarone D, Gilchrist G, McCusker M, O'Brien K, Dunn J, Scott J, Hope V. 'Care and Prevent': rationale for investigating skin and soft tissue infections and AA amyloidosis among people who inject drugs in London. *Harm Reduct J*. 2018;15(1):23.
19. National Institute for Health and Care Excellence (NICE). 'Guidance: methadone and buprenorphine for the management of opioid dependence'. 2007. Available from: https://www.nice.org.uk/guidance/ta114/resources/methadone-and-buprenorphine-for-the-management-of-opioid-dependence-pdf-82598072878789
20. Public Health England. Guidance: Part 1: introducing opioid substitution treatment (OST). 2021. Available from: https://www.gov.uk/government/publications/opioid-substitution-treatment-guide-for-keyworkers/part-1-introducing-opioid-substitution-treatment-ost
21. Department of Health and Social Care. 2017. Drug misuse and dependence UK guidelines on clinical management. Available from: https://www.gov.uk/government/publications/drug-misuse-and-dependence-uk-guidelines-on-clinical-management
22. SmPC Methadone 1mg/ml oral solution sugar-free. Available from: https://www.medicines.org.uk/emc/product/3702/smpc#gref
23. BNF Guidelines for methadone. Available from: https://bnf.nice.org.uk/drugs/methadone-hydrochloride/
24. Faculty of Pain Medicine. 'Substance misuse: acute pain management'. (no date). Available from: https://fpm.ac.uk/opioids-aware-opioids-addiction/substance-misuse-acute-pain-management
25. Wesson DR, Ling W. The clinical opiate withdrawal scale (COWS). *J Psychoact Drugs*. 2003;35(2):253-9.

15.0 Flow chart for OST prescribing

1. Patient assessment for opioid dependence

- Establish use: patient history of drug use (community OST prescription and/or heroin use)
- Establish tolerance: medication, formulation, dose, frequency of pick-up, supervised/unsupervised (OST); amount, frequency, route, and duration of use (other opiates)
- Physical examination for withdrawal symptoms, e.g. using the Clinical Opioid Withdrawal Scale (Appendix 3)

NB: Do not prescribe OST if contraindications: head injury, acute respiratory depression, coma (see BNF).

Evidence of opioid dependence?

No → Do not prescribe OST; monitor for symptoms of withdrawal

Yes → On a community OST prescription?

No → go to 3. Initiating OST in hospital

Yes → Dose confirmed (prescriber/pharmacist); last consumption within 3 days (patient if unsupervised Rx / pharmacist if supervised Rx)

No → go to 3. Initiating OST in hospital

Yes → 2. Continuing community OST prescription

2. Continuing community OST prescription

- Rx naloxone PRN
- Inform CDTS of admission; discuss changes in dosing and discharge planning

Day one

- Rx usual dose once daily (BD if cautions or if preferred by patient)
- Monitor withdrawal symptoms 4-hourly
- [Methadone only]: if withdrawal symptoms persist, Rx additional 5-10mg PRN methadone (max. 10mg/day) to cover on-top opioid use

Day two onwards

- [Methadone only] Adjust usual dose to include any additional methadone and convert into daily dose; max. weekly dose increase 30mg

For patients whose dose cannot be promptly confirmed: See Box 3; once dose confirmed, Rx any additional OST needed to meet total usual dose; Rx usual daily dose from following day

3.Initiating OST in hospital

- Inform local CDTS to facilitate referral on discharge

**Methadone (1mg/1ml)**	**Buprenorphine**
Day one<br>• Rx naloxone PRN<br>• Rx initial dose 20mg (split dosing if cautions); consider 30mg with skilled clinical judgement<br>• Rx additional dose 10mg PRN 4-hourly up to 40mg total day-one dose if withdrawal symptoms persist<br>• If withdrawal symptoms persist 4 hours after 40mg dose, consider additional 10mg dose 4-hourly up to max. 60mg total day-one dose under expert supervision (p. 12)	Day one<br>• Rx naloxone PRN<br>• Rx 4mg buprenorphine (NB: only administer initial dose buprenorphine when withdrawal symptoms are present)<br>• Monitor for withdrawal symptoms 4-hourly; if withdrawal symptoms persist, Rx additional 2mg PRN (up to 8mg total day-one dose)
Day two onwards<br>• Convert total day-one dose into daily prescription and Rx in divided doses (BD)<br>• Monitor for withdrawal symptoms; Rx additional 10mg/day every other day (maximum 30mg increase over seven days); If day-one dose ≤40mg, can increase dose by 10mg on day two	Day two onwards<br>• Convert total day-one dose into daily prescription and Rx once daily<br>• Monitor for withdrawal symptoms; Rx additional 4mg dose 4-hourly if required, up to 16mg day-two dose

Monitor all patients for signs of opioid toxicity 4 hours after each dose and then as per NEWS

- If RR<12, oxygen saturation below target, or if reduced level of consciousness, call ward doctor urgently

4. Acute pain management (see page 12)

5. Discharge

**Patients admitted on community Rx**	**Patients initiated on OST in hospital**
• Rx OST on day of discharge; Rx TTA naloxone<br>• Contact CDTS to continue/re-start community Rx<br>• Where agreed with CDTS, Rx TTA OST if discharged out-of-hours to prevent break in OST	• Rx OST on day of discharge; Rx TTA naloxone<br>• Arrange urgent appt. with CDTS<br>• Arrange bridging prescription to prevent break in OST if same-day appt. unavailable

16.0 MEDL guideline

Medl

NHS
University College London Hospitals
NHS Foundation Trust

Prevention and treatment of opioid withdrawal in hospital

See Full Guideline for further information
See Flowsheet (appendix 1) and Clinical Opiate Withdrawal Scale (COWS) (appendix 2)

DIAGNOSIS AND CAUSES

Use ***.methadoneOST*** or ***.buprenorphineOST***

1. Establish opioid dependency
- Community opioid substitution therapy (OST) Rx
- Regular heroin use (frequency, amount, route)
- History of opioid withdrawal symptoms

2. Conduct physical examination
- Opioid withdrawal symptoms using clinically validated scoring tool, e.g., **COWS** (**Appendix 2**)
- Polysubstance use (NB: alcohol withdrawal is a medical emergency; see local guidance [LINK])

3. For patients on community Rx:
- Confirm medication, formulation, current dose, and whether consumption is supervised (community pharmacist/prescriber); confirm date of last consumption (pharmacist if supervised/patient if unsupervised)

NB: Re-titrate OST if last consumption reported as 3+ days from date of hospital admission

REQUEST

Monitor all patients for opioid toxicity four hours after each dose and then as per NEWS
- If RR<12, oxygen saturation below target, or reduced level of consciousness: withhold OST
- **If unresponsive: administer naloxone**

ADVICE, REVIEWS & REFERRALS

- Inform Drug and Alcohol Liaison CNS of all patients prescribed OST in hospital: 07415331353
- Liaise with Community Drug Treatment (CDTS) service for all patients prescribed OST in hospital
- See section in full guideline on acute pain management

DISCHARGE

- Administer full dose OST on day of discharge
- Prescribe **TTA naloxone (Prenoxad)**
- **Patients admitted on community Rx**: Arrange continuation of OST Rx with CDTS (*OOH bridging supply not permitted if not pre-agreed with CDTS*)
- For patients initiated on OST in hospital: Arrange urgent appointment with CDTS for day of discharge

MEDL GUIDELINE DETAILS
Authors: iHOST team (M Harris, A Holland, J Scott & M Wickremsinhe), M Brown, J Norman **MEDL Editor:** M George.
Specialist: Drug and Alcohol Team **Pharmacist:** Agnes Niemet
CGC approval: 11/10/22 Review date: 11/10/25

TREAT

- **Rx naloxone PRN for all patients on OST**
- NB: Do not prescribe OST if contraindications: head injury, acute respiratory depression, coma (see BNF)
- You **DO NOT** have to send a urinary Tox Screen before prescribing OST

Continuing community Rx

Use ***.methadoneOST*** or ***.buprenorphineOST***
- Rx usual dose once daily (BD dosing if cautions, patient preference, or to enable pain management)
- ***For patients on methadone***: monitor for symptoms of withdrawal; if withdrawal symptoms persist, prescribe 5-10mg methadone PRN; max. daily dose increase 10mg, max. weekly dose increase 30mg

Initiating/re-titrating methadone (1mg/1ml)

(use ***.methadoneOST*** smartphrase)

DAY ONE
- Rx 20mg starting dose methadone
- Monitor for symptoms of withdrawal 4-hourly
- Rx additional 10mg PRN methadone 4-hourly up to 40mg total day-one dose
- If withdrawal symptoms persist, prescribe up to 60mg total day-one dose **under expert supervision**

DAY TWO ONWARDS
- Convert total day-one dose into daily prescription and Rx in divided doses (BD)
- Monitor for withdrawal symptoms; if withdrawal symptoms persist:
  - Increase dose by up to 10mg PRN every other day (max. weekly dose increase of 30mg over day-one dose); If day-one dose ≤40mg, can increase dose by up to 10mg on day two

Initiating/re-titrating buprenorphine

(use ***.buprenorphineOST*** smartphrase)

DAY ONE
- **NB: Only administer buprenorphine when withdrawal symptoms are present**
- Rx 4mg buprenorphine
- Monitor for withdrawal symptoms 4-hourly; if withdrawal symptoms persist, Rx additional 2mg PRN (up to 8mg total day-one dose)

DAY TWO ONWARDS
- Convert total day-one dose into daily prescription and Rx once daily
- Monitor for withdrawal symptoms; Rx additional 4mg dose 4-hourly if required, up to 8mg total PRN (max. 16mg total day-two dose)

`

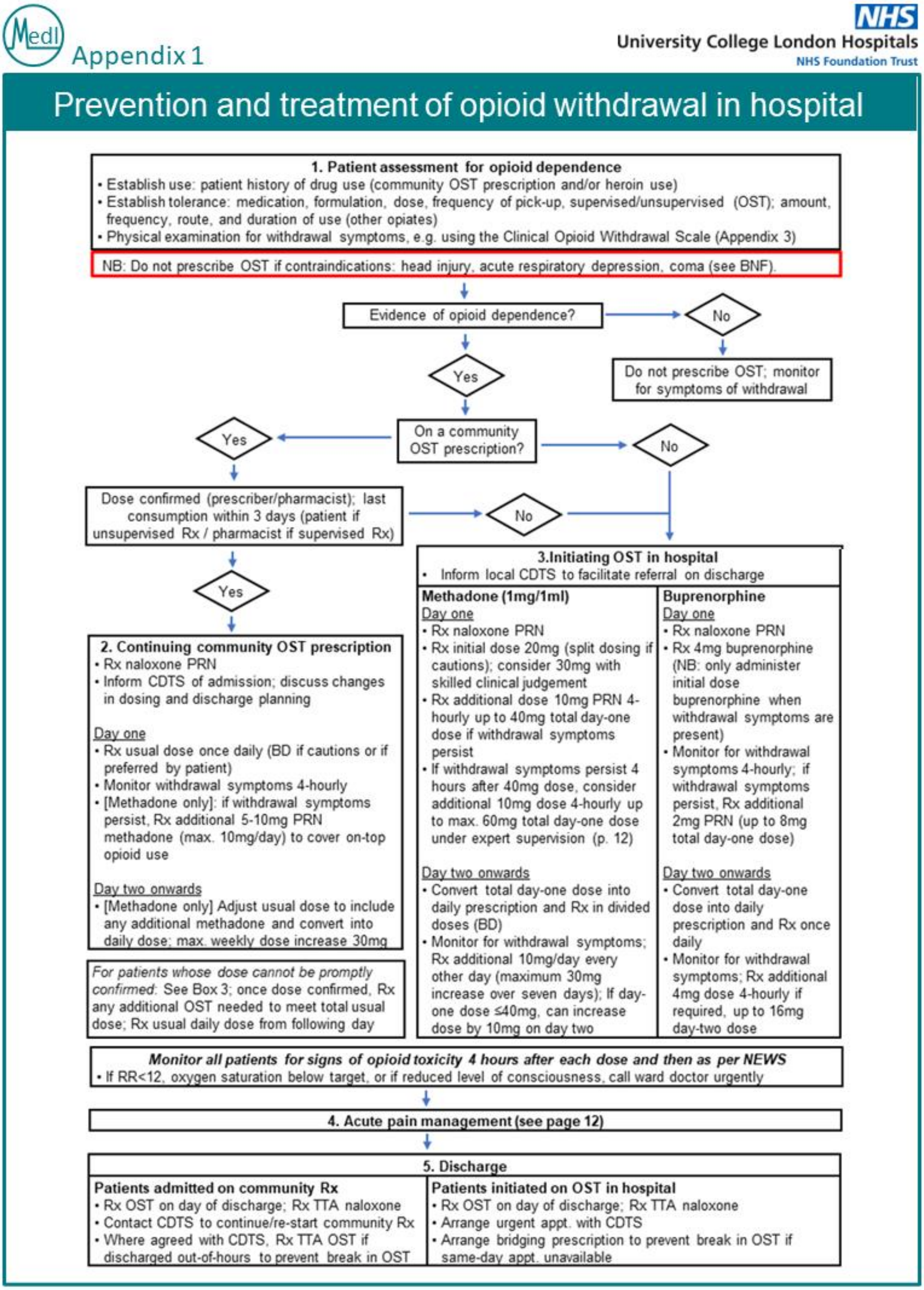

Medl
Appendix 1
NHS
University College London Hospitals
NHS Foundation Trust
Prevention and treatment of opioid withdrawal in hospital
1. Patient assessment for opioid dependence
• Establish use: patient history of drug use (community OST prescription and/or heroin use)
• Establish tolerance: medication, formulation, dose, frequency of pick-up, supervised/unsupervised (OST); amount, frequency, route, and duration of use (other opiates)
• Physical examination for withdrawal symptoms, e.g. using the Clinical Opioid Withdrawal Scale (Appendix 3)
NB: Do not prescribe OST if contraindications: head injury, acute respiratory depression, coma (see BNF).
Evidence of opioid dependence?
No
Do not prescribe OST; monitor for symptoms of withdrawal
Yes
On a community OST prescription?
Yes
No
Dose confirmed (prescriber/pharmacist); last consumption within 3 days (patient if unsupervised Rx / pharmacist if supervised Rx)
No
Yes
2. Continuing community OST prescription
• Rx naloxone PRN
• Inform CDTS of admission; discuss changes in dosing and discharge planning
Day one
• Rx usual dose once daily (BD if cautions or if preferred by patient)
• Monitor withdrawal symptoms 4-hourly
• [Methadone only]: if withdrawal symptoms persist, Rx additional 5-10mg PRN methadone (max. 10mg/day) to cover on-top opioid use
Day two onwards
• [Methadone only] Adjust usual dose to include any additional methadone and convert into daily dose; max. weekly dose increase 30mg
For patients whose dose cannot be promptly confirmed: See Box 3; once dose confirmed, Rx any additional OST needed to meet total usual dose; Rx usual daily dose from following day
3.Initiating OST in hospital
• Inform local CDTS to facilitate referral on discharge
Methadone (1mg/1ml)
Day one
• Rx naloxone PRN
• Rx initial dose 20mg (split dosing if cautions); consider 30mg with skilled clinical judgement
• Rx additional dose 10mg PRN 4-hourly up to 40mg total day-one dose if withdrawal symptoms persist
• If withdrawal symptoms persist 4 hours after 40mg dose, consider additional 10mg dose 4-hourly up to max. 60mg total day-one dose under expert supervision (p. 12)
Day two onwards
• Convert total day-one dose into daily prescription and Rx in divided doses (BD)
• Monitor for withdrawal symptoms; Rx additional 10mg/day every other day (maximum 30mg increase over seven days); If day-one dose ≤40mg, can increase dose by 10mg on day two
Buprenorphine
Day one
• Rx naloxone PRN
• Rx 4mg buprenorphine (NB: only administer initial dose buprenorphine when withdrawal symptoms are present)
• Monitor for withdrawal symptoms 4-hourly; if withdrawal symptoms persist, Rx additional 2mg PRN (up to 8mg total day-one dose)
Day two onwards
• Convert total day-one dose into daily prescription and Rx once daily
• Monitor for withdrawal symptoms; Rx additional 4mg dose 4-hourly if required, up to 16mg day-two dose
Monitor all patients for signs of opioid toxicity 4 hours after each dose and then as per NEWS
• If RR<12, oxygen saturation below target, or if reduced level of consciousness, call ward doctor urgently
4. Acute pain management (see page 12)
5. Discharge
Patients admitted on community Rx
• Rx OST on day of discharge; Rx TTA naloxone
• Contact CDTS to continue/re-start community Rx
• Where agreed with CDTS, Rx TTA OST if discharged out-of-hours to prevent break in OST
Patients initiated on OST in hospital
• Rx OST on day of discharge; Rx TTA naloxone
• Arrange urgent appt. with CDTS
• Arrange bridging prescription to prevent break in OST if same-day appt. unavailable

Medl Appendix 2

NHS University College London Hospitals NHS Foundation Trust

Clinical Opiate Withdrawal Scale (COWS)

Resting pulse rate (beats/minute)
0 - Pulse rate 80 or below
1 - Pulse rate 81-100
2 - Pulse rate 101 -120
4 - Pulse rate greater than 120

Sweating (over past ½ hour, not accounted for by room temperature or client activity)
0 - No chills of flushing
1 - Patient report of chills or flushing
2- Flushing or observable moistness on face
3 - Beads of sweat on brow or face
4 - Sweat streaming off face

Restlessness (observation during assessment)
0 - Able to sit still
1 - Reports difficulty sitting still, but is able to do so
3 - Frequent shifting or extraneous movements of legs/arms
5 - Unable to sit still for more than a few seconds

Pupil dilation
0 - Pupils pinned or normal size for room light
1 - Pupils possibly larger than normal for room light
2 - Pupils moderately dilated
5 - Pupils so dilated that only the rim of the iris is visible

Bone or joint aches (not attributable to other health conditions or presenting complaint)
0 - Not present
1 - Mild diffuse discomfort
2 - Client reports severe diffuse aching of joints/muscles
4 - Client is rubbing joints or muscles and is unable to sit still because of discomfort

Rhinorrhoea or lacrimation (not accounted for by cold symptoms or allergies)
0 - Not present
1 - Nasal stuffiness or unusually moist eyes
2 - Nose running or tearing
4 - Nose constantly running or tears streaming down cheeks

Vomiting, nausea, and/or diarrhoea (over last ½ hour)
0 - No GI symptoms
1 - Stomach cramps
2 - Nausea or loose stool
3 - Vomiting or diarrhoea
5 - Multiple episodes of diarrhoea or vomiting

Tremor (observation of outstretched hands)
0 - No tremor
1 - Tremors can be felt, but not observed
2 - Slight tremors observable
4 - Gross tremor or muscle twitching

Yawning
0 - No yawning
1 - Yawning once or twice during assessment
2 - Yawning three or more times during assessment
4 - Yawning several times per minute

Gooseflesh skin
0 - Skin is smooth
3 - Piloerection of skin can be felt or hairs standing up on arms
5 - Prominent piloerection

Scoring: "Mild"— 5 to 12; "Moderate"—13 to 24; "Moderately severe"—25 to 36; "Severe"—more than 36

This MEDL guideline is intended to support decision making by trained doctors and must be interpreted appropriately in the clinical context. It covers management for **adult patients only**. The authors cannot be held responsible for their use. Only current on date printed, refer to myUCLH for definitive version.

Appendix 1: Comparison of buprenorphine and methadone for withdrawal management in acute hospital settings

**Methadone**—*full opioid agonist*	**Buprenorphine—***partial opioid agonist*
Clinical considerations and management<br>• No ceiling effect, which increases risk of overdose (compared with buprenorphine)<br>• As a full agonist, does not significantly affect pain management on top of OST	Clinical considerations and management<br>• Ceiling effect reduces risk of overdose<br>• As a partial agonist, can interfere with analgesia; care needed when prescribing pain management on top of OST
Patient experience<br>• Does not interfere with euphoric action of other opioids used on top	Patient experience<br>• Clearer head compared to methadone<br>• Blocks euphoric action of other opioids used on top
Contraindications<br>• Acute respiratory depression<br>• Head injury<br>• Raised intracranial pressure<br>• Risk of paralytic ileus<br>• Coma<br>• Phaeochromocytoma<br><br>Cautions<br>• Decompensated liver disease<br>• Severe renal impairment<br>• Risk factors for QT-interval prolongation<br>• Impaired respiratory function from COPD or asthma<br>• Concomitant use of other CNS depressants (e.g., benzodiazepines) or gabapentinoids<br><br>*For full list of contraindications and cautions, see BNF Guidelines*	Contraindications<br>• Acute respiratory depression<br>• Head injury<br>• Raised intracranial pressure<br>• Risk of paralytic ileus<br>• Coma<br><br>Cautions<br>• Decompensated liver disease<br>• Impaired respiratory function from COPD or asthma<br>• Concomitant use of other CNS depressants (e.g., benzodiazepines) or gabapentinoids<br><br>*For full list of contraindications and cautions, see BNF Guidelines*
Safety considerations<br>• Peak plasma levels occur 4 to 6 hours after dose administration<br>• Delayed toxicity due to long and variable half-life (13 to 47 hours)<br>• Cumulative effect increases risk of overdose<br>• Increased risk of overdose when used with other CNS depressants	Safety considerations<br>• Can precipitate withdrawal<br>• Increased risk of overdose when used with other CNS depressants
Therapeutic doses in the community<br>Range from 60-120mg daily (though doses on either side of this range are routinely prescribed in community settings)	Therapeutic doses in the community<br>• Range from 12-24mg daily, with a maximum of 32mg for sublingual formulations

Appendix 2: Taking a comprehensive drug history

People who use drugs may not wish to disclose details about their drug use. Clear, empathetic, and non-judgemental communication is critical to eliciting a thorough and accurate drug history to enable provision of the best medical care. Asking the patient about their drug use in judgemental ways may increase felt stigma and could compromise the therapeutic relationship. When taking a drug history, clinicians should offer brief explanations as to why the information elicited is important to the patient's care.

People who use drugs may consider some terms stigmatising. While patients may describe themselves as a 'user' or 'addict', it is best practice to avoid using these terms. Person-centred language (i.e., 'people who use drugs') is preferred. Similarly, 'drug use' or 'drug dependence' may be preferred to terms like 'drug misuse', 'drug abuse', or 'drug use disorder'.

<u>Establish history of opioid use</u>

For example: *"It is important that we understand what doses of opioids, like methadone and heroin, you take in the community, so we can prescribe enough medication to keep you comfortable, whilst not prescribing too much, which could cause you to overdose."*

For patients receiving OST in the community:

- Formulation
- Dose (stabilised doses in the community tend to range from 60mg to 120mg, but doses on either side of this range are routinely used)
- Community prescriber (e.g., GP, community drug treatment service)
- Community pharmacy
- Frequency of pick-up (every day, weekly, twice weekly)
- Supervised or unsupervised consumption
- Last dose consumed
- Doses brought into hospital
- Use of heroin or other opioids on top of OST

[NOTE: After gathering this information from the patient directly, confirm dose and last consumption (if patient is on supervised consumption) with the community pharmacist and/or community prescriber. If the prescriber or pharmacist are unavailable (i.e., if the patient presents out-of-hours), alternative sources of dose confirmation may include:

- Key worker (some services have an out-of-hours contact number)
- Current, labelled OST medication bottles/prescriptions brought to hospital by patient

For patients not in receipt of a community OST prescription:

- Use of heroin or other illicit opioids
- Duration of use ('When did you first start using?')
- Frequency of use ('How often do you use?')
- Last dose consumed

Current symptoms of opioid withdrawal (See Section 10.2.2)

- Consider assessment using COWS (Appendix 1).

Past experiences of opioid withdrawal symptoms

Establish history of use of any other drugs

For example: "*In order to help you feel comfortable, and reduce risks of any treatment while in hospital, it is important for us to understand what other drugs you are regularly taking. These other drugs could interact with medications that we might need to prescribe for you, and withdrawal from some drugs, like alcohol and benzodiazepines, can be dangerous if not appropriately treated*"

N.B. Severe alcohol withdrawal is a medical emergency—see local guidance [LINK].

Alcohol use, dependence, and/or withdrawal
- Units consumed per week
- Previous experience of alcohol withdrawal
- Current symptoms of alcohol withdrawal (e.g., tremors, sweating, tachycardia, nausea, vomiting, headaches, restlessness, insomnia)
- Current symptoms of delirium tremens (e.g., disorientation, hallucinations, severe agitation, hypertension, pyrexia, seizures)

Benzodiazepine use, dependence, and/or withdrawal
- Use, frequency, duration, dose
- Previous experience of benzodiazepine withdrawal
- Current symptoms of benzodiazepine withdrawal (e.g. tremors, sweating, nausea, vomiting, headaches, lack of appetite, hallucinations, seizures

Nicotine use
- Consider nicotine patch

Other illicit drug use
For each drug:
- Duration of use
- Frequency of use
- Dose
- Route of administration

Additional health needs
Patients with opioid dependence are often underserved by healthcare services. As such, attendance at hospital may provide an opportunity to facilitate additional preventative and treatment interventions. After managing the acute presentation, consider:
- Treatment of complications of drug use
- Testing for bloodborne viruses (HIV, hepatitis B, hepatitis C) and referral for treatment
- Immunisation for hepatitis A, hepatitis B, and tetanus if not up to date (with advice on flue and pneumococcal vaccines, particularly for patients who are older or have respiratory comorbidities)
- Cervical cancer screening
- Access to need and syringe programmes
- Access to takeaway naloxone
- Information about/referral to NHS dental services
- Contraception advice and provision

Appendix 3: Clinical Opioid Withdrawal Scale (COWS)[25]

Symptoms	Time points					
	0hrs	4hrs	8hrs	12hrs	24hrs	48hrs
**Resting pulse rate (beats/minute)**<br>0 - Pulse rate 80 or below<br>1 - Pulse rate 81-100<br>2 - Pulse rate 101 -120<br>4 - Pulse rate greater than 120						
**Sweating (over past ½ hour, not accounted for by room temperature or client activity)**<br>0 - No chills of flushing<br>1 - Patient report of chills or flushing<br>2- Flushing or observable moistness on face<br>3 - Beads of sweat on brow or face<br>4 - Sweat streaming off face						
**Restlessness (observation during assessment)**<br>0 - Able to sit still<br>1 - Reports difficulty sitting still, but is able to do so<br>3 - Frequent shifting or extraneous movements of legs/arms<br>5 - Unable to sit still for more than a few seconds						
**Pupil dilation**<br>0 - Pupils pinned or normal size for room light<br>1 - Pupils possibly larger than normal for room light<br>2 - Pupils moderately dilated<br>5 - Pupils so dilated that only the rim of the iris is visible						
**Bone or joint aches (not attributable to other health conditions or presenting complaint)**<br>0 - Not present<br>1 - Mild diffuse discomfort<br>2 - Client reports severe diffuse aching of joints/muscles<br>4 - Client is rubbing joints or muscles and is unable to sit still because of discomfort						
**Rhinorrhoea or lacrimation (not accounted for by cold symptoms or allergies)**<br>0 - Not present<br>1 - Nasal stuffiness or unusually moist eyes<br>2 - Nose running or tearing<br>4 - Nose constantly running or tears streaming down cheeks						
**Vomiting, nausea, and/or diarrhoea (over last ½ hour)**<br>0 - No GI symptoms<br>1 - Stomach cramps<br>2 - Nausea or loose stool<br>3 - Vomiting or diarrhoea<br>5 - Multiple episodes of diarrhoea or vomiting						
**Tremor (observation of outstretched hands)**<br>0 - No tremor<br>1 - Tremors can be felt, but not observed<br>2 - Slight tremors observable<br>4 - Gross tremor or muscle twitching						
**Yawning**<br>0 - No yawning<br>1 - Yawning once or twice during assessment<br>2 - Yawning three or more times during assessment<br>4 - Yawning several times per minute						
**Gooseflesh skin**<br>0 - Skin is smooth<br>3 - Piloerection of skin can be felt or hairs standing up on arms<br>5 - Prominent piloerection						
**TOTAL**						

Scoring: "Mild"— 5 to 12; "Moderate"—13 to 24; "Moderately severe"—25 to 36; "Severe"—more than 36

Appendix 4: Resources for training patients to use naloxone

Prenoxad® patient leaflet

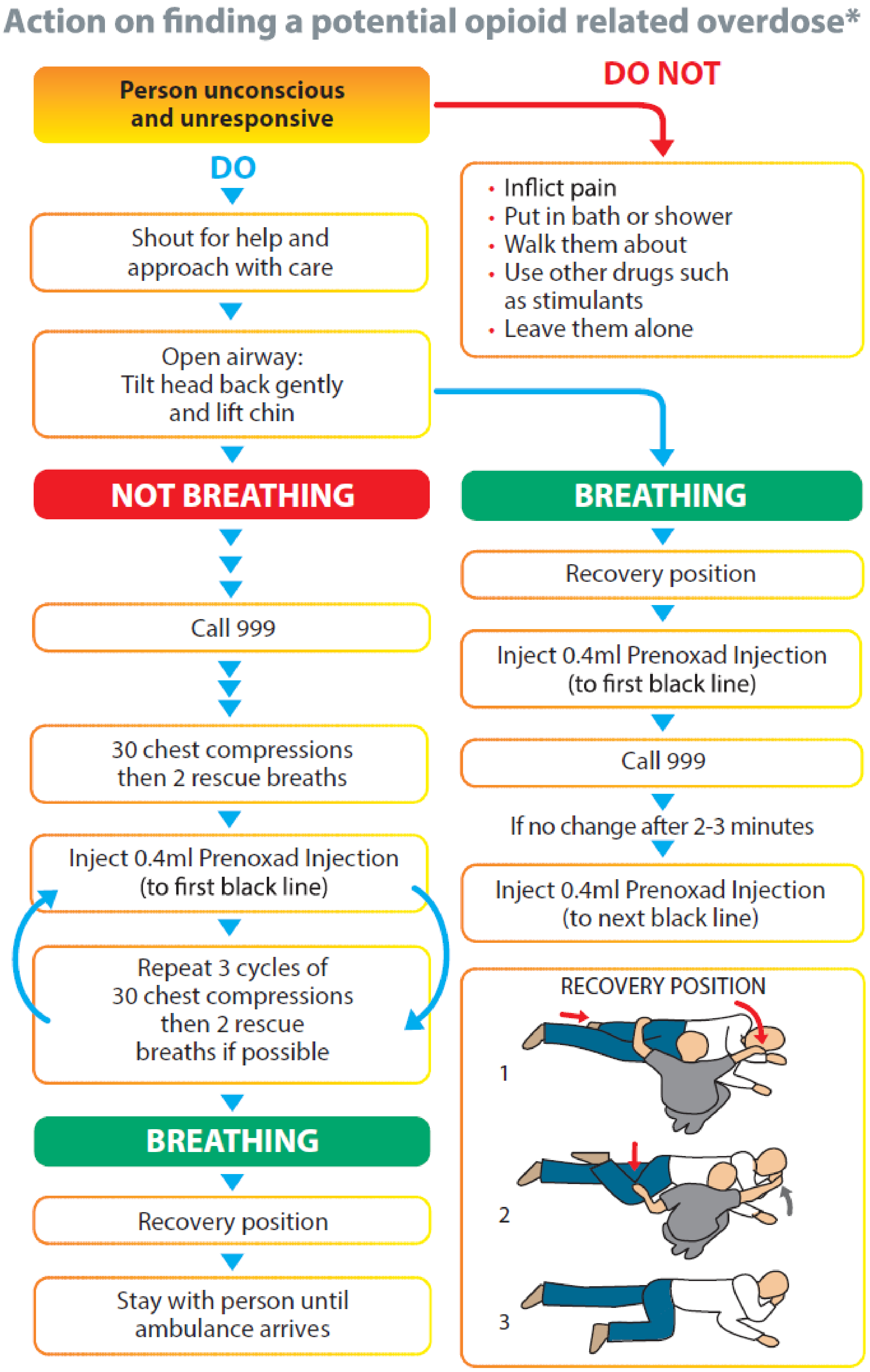

Description of how to assemble and use Prenoxad® Injection

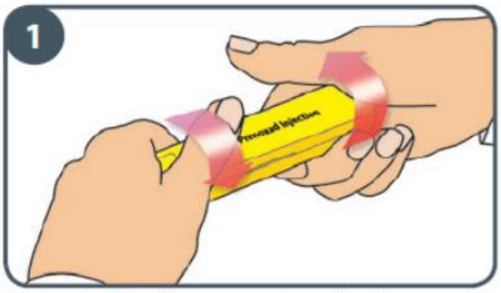

Remove the clear film wrapping by pulling the tear strip on the side of the box. Twist the outer plastic box as shown to break the tamper evident seals and open.

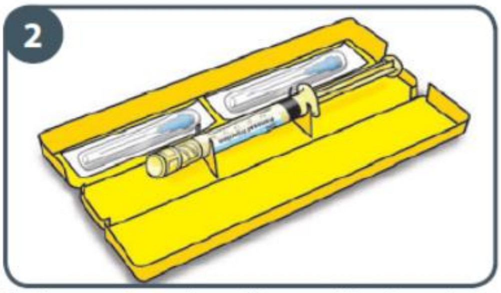

The box contains 1 syringe of Prenoxad Injection and two needles.

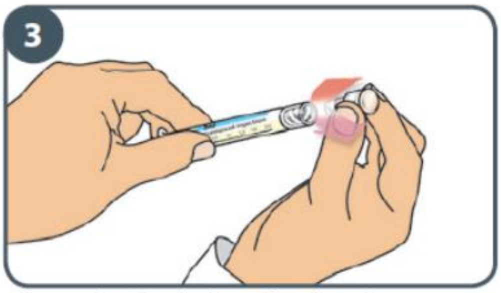

Unscrew the clear plastic top from the syringe.

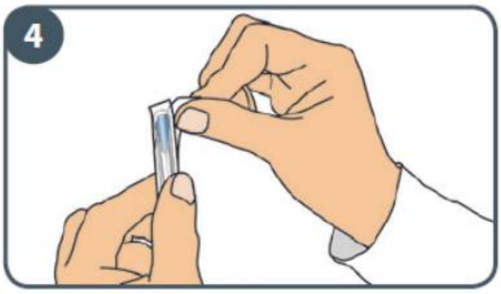

Peel back the backing paper from the needle packet and remove the needle in its protective sheath.

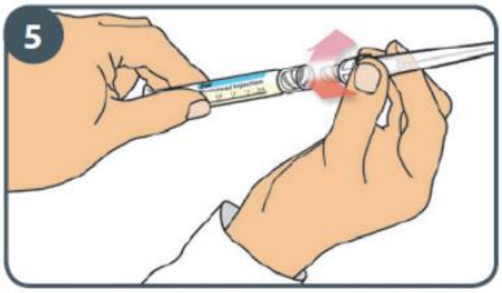

With the needle still in its sheath, screw the blue fitting on to the syringe.

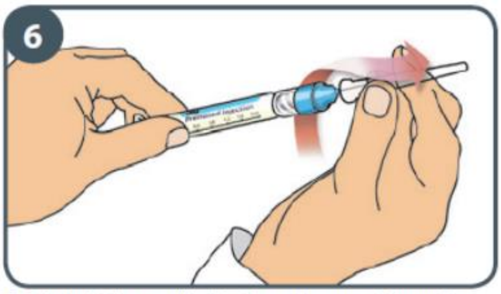

Gently twist the needle sheath and remove it from the syringe.

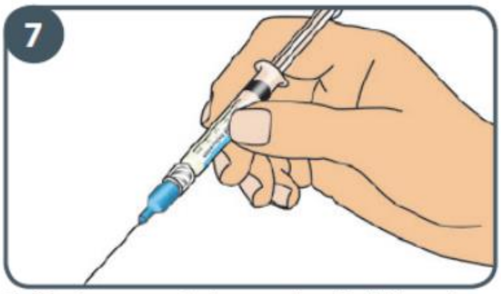

To inject someone who has overdosed, hold the syringe like a pen

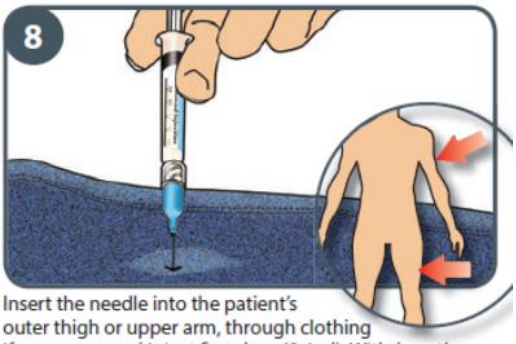

Insert the needle into the patient's outer thigh or upper arm, through clothing if necessary, and inject first dose (0.4ml). Withdraw the needle and syringe after each dose.

Training checklist (adapted from the Scottish Drugs Forum)

Background	
**The most common drugs identified in a drug-related death** (heroin, methadone, diazepam and alcohol – all CNS depressant drugs) **and the physical effects these drugs have** (slow, shallow, irregular breathing; slow heart rate; feeling less alert; unconsciousness; poor memory; not feeling pain; lower body temperature)	
**The main causes of drug overdose** (low tolerance, polydrug use, using too much, using alone, injecting drug use, purity levels)	
**High risk times** (release from prison, leaving rehabilitation or hospital, recent detox, recent relapse, poor physical or mental health, recent life events, cash windfall, longer-term user, festive periods, weekends or holidays)	
**The signs and symptoms of suspected opiate overdose** (pinpoint pupils; breathing problems; skin/lip colour; no response to noise or touch; loss of consciousness)	
**The common myths** ("give other drugs e.g. stimulants", "put person in the bath or shower", "walk person around", "leave person on own", "shake or shock person")	
Process	
**Knows to call 999 immediately**	
**Knows about the recovery position** (person on side, airway open)	
**Knows about rescue breathing and CPR** (30 compressions, 2 breaths – one cycle of BLS)	
**Knows what the kit contains** (syringe with naloxone and two needles; the syringe contains five 0.4ml doses; if needed, all five doses can be administered according to the dosing schedule using the same needle; if doses are not used, dispose of entire kit safely)	
**Knows when and how to administer naloxone** (dose: 0.4 mls into outer thigh muscle via clothing; assembly of syringe):<br>• Unconscious but breathing – administer when in recovery position and then every 2-3 minutes<br>• Unconscious but NOT breathing – administer after one cycle of BLS then after every three cycles of BLS	
**Knows that naloxone is short acting** (the effects of naloxone wear off after 20-30 minutes, meaning it is possible that overdose may return)	
**Knows the importance of staying with the person** (do not let the person use any other drugs if they gain consciousness)	
**Knows how to safely dispose of the used syringe and needle** (the used syringe and needle should be placed back in the plastic box and then given to the ambulance crew on arrival for safe disposal)	

Link to Prenoxad® training video (Exchange Supplies)

https://training.exchangesupplies.org/Prenoxad_access-launch

Appendix 5: Key guidelines and resources

- BNF Guidelines for methadone: https://bnf.nice.org.uk/drugs/methadone-hydrochloride/
- BNF Guidelines for buprenorphine: https://bnf.nice.org.uk/drugs/buprenorphine/
- NICE Guidance on methadone and buprenorphine for the management of opioid dependence: https://www.nice.org.uk/guidance/TA114
- Department of Health and Social Care. *Drug misuse and dependence UK guidelines on clinical management*: https://www.gov.uk/government/publications/drug-misuse-and-dependence-uk-guidelines-on-clinical-management
- Faculty of Pain Medicine recommendations on acute pain management for patients on OST: https://fpm.ac.uk/opioids-aware-opioids-addiction/substance-misuse-acute-pain-management
